# Sub-10-nm-sized Au@Au*_x_*Ir_1−_*_x_* metal-core/alloy-shell nanoparticles as highly durable catalysts for acidic water splitting

**DOI:** 10.1093/nsr/nwae056

**Published:** 2024-02-07

**Authors:** Huimin Wang, Zhe-ning Chen, Yuanyuan Wang, Dongshuang Wu, Minna Cao, Fanfei Sun, Rong Cao

**Affiliations:** State Key Laboratory of Structural Chemistry, Fujian Institute of Research on the Structure of Matter, Chinese Academy of Sciences, Fuzhou 350002, China; University of Chinese Academy of Sciences, Beijing 100049, China; State Key Laboratory of Structural Chemistry, Fujian Institute of Research on the Structure of Matter, Chinese Academy of Sciences, Fuzhou 350002, China; School of Materials Science and Engineering, Nanyang Technological University, Singapore 639798, Singapore; School of Materials Science and Engineering, Nanyang Technological University, Singapore 639798, Singapore; State Key Laboratory of Structural Chemistry, Fujian Institute of Research on the Structure of Matter, Chinese Academy of Sciences, Fuzhou 350002, China; University of Chinese Academy of Sciences, Beijing 100049, China; Shanghai Synchrotron Radiation Facility, Shanghai Institute of Applied Physics, Chinese Academy of Sciences, Shanghai 201204, China; State Key Laboratory of Structural Chemistry, Fujian Institute of Research on the Structure of Matter, Chinese Academy of Sciences, Fuzhou 350002, China; University of Chinese Academy of Sciences, Beijing 100049, China; Fujian Science & Technology Innovation Laboratory for Optoelectronic Information of China, Fuzhou 350108, China

**Keywords:** controllable synthesis of surface IrO*_x_*, oxygen evolution reaction, acidic water splitting, catalytic stability, transition metal catalysts

## Abstract

The absence of efficient and durable catalysts for oxygen evolution reaction (OER) is the main obstacle to hydrogen production through water splitting in an acidic electrolyte. Here, we report a controllable synthesis method of surface IrO*_x_* with changing Au/Ir compositions by constructing a range of sub-10-nm-sized core-shell nanocatalysts composed of an Au core and Au*_x_*Ir_1−_*_x_* alloy shell. In particular, Au@Au_0.43_Ir_0.57_ exhibits 4.5 times higher intrinsic OER activity than that of the commercial Ir/C. Synchrotron X-ray-based spectroscopies, electron microscopy and density functional theory calculations revealed a balanced binding of reaction intermediates with enhanced activity. The water-splitting cell using a load of 0.02 mg_Ir_/cm^2^ of Au@Au_0.43_Ir_0.57_ as both anode and cathode can reach 10 mA/cm^2^ at 1.52 V and maintain activity for at least 194 h, which is better than the cell using the commercial couple Ir/C‖Pt/C (1.63 V, 0.2 h).

## Teaser

Surface active and durable IrOx is controlled by the electronic interaction between Au and Ir, which can realize a balance for binding of different intermediates and accelerate the water splitting kinetics.

## INTRODUCTION

The development of proton-exchange-membrane water electrolyzers (PEMWEs) leads to the production of hydrogen in a more environmentally friendly way [1,2]. However, the oxygen evolution reaction (OER) at the anode is more challenging with the four-electron process, compared to the cathodic hydrogen evolution reaction (HER) with two-electron transferring [3,4]. Large overpotential (activity aspect) is required for OER due to the slow reaction kinetics even using the OER catalysts; however, such a reaction condition will greatly accelerate the corrosion and dissolution of catalysts (durability aspect) especially in the strong acidic electrolyte of PEMWEs [5–8]. Thus, activity and durability of catalysts are often correlated inversely [9,10]. Currently, thermodynamically stable rutile IrO_2_ is the only benchmarking catalyst for OER in acidic media, with considerable stability, but it also requires large overpotential to achieve a decent current density (>300 mV at 10 mA/cm^2^ geometric activity) [11–13]. Although Ir-based metal nanoparticles (NPs) exhibit superior OER activity to IrO_2_ (generally with more than 40 mV differences in the overpotential at 10 mA/cm^2^) due to the formation of (hydro)oxides species with uncoordinated O/Ir atoms (denoted as IrO*_x_*) [14] during the electrochemical process [13,15,16], it is believed that IrO*_x_* is less stable than IrO_2_ [17]. Alloying Ir with a second metal, such as Ni, Fe, V or Ru, can improve the activity and/or durability due to the electronic effect or steric effect [1,8–23]. However, these metals can be easily leached, even at a potential much lower than OER in acidic media, leading to significant degradation in catalytic performance. Currently, synthesizing and stabilizing surface IrO*_x_* in a controlled manner remains a challenging task.

Au-Ir alloy might have higher durability for OER since Au rarely dissolves in acid even at 1.8 V [5,24]. Quite recently, we have shown that the surface of Ir goes through partial oxidation with Au modification, which suggests the possibility of controllable formation of IrO*_x_* in an Au-Ir binary structure [24]. However, Au and Ir are not miscible in the phase diagram and Ir tends to form clusters individually [25,26]. Thus, constructing homogenous Au-Ir nanostructures is still a challenge [27,28].

Herein, we report a core-shell structure with an Au core and Au-Ir shell with tunable Ir composition. All these catalysts showed higher OER activity than the benchmarking IrO_2_. In particular, Au@Au_0.43_Ir_0.57_ exhibits optimal performance. The intrinsic/mass activities of Au@Au_0.43_Ir_0.57_ are 7.77 mA/cm^2^ and 3.40 A/mg_Ir_ (at overpotential (*η*) = 300 mV), which are 4.5 (7.7) times higher than those of a commercial Ir catalyst and the most active catalysts reported in acidic media for OER. Au@Au_0.43_Ir_0.57_ also shows higher HER activity with 23 mV at 10 mA/cm^2^, compared to Pt/C (33 mV). Synchrotron-radiation-based X-ray absorption fine structure spectroscopy (XAFS) and in-depth X-ray photoelectron spectra (XPS) revealed that surface IrO*_x_* can be controlled by changing the Au/Ir compositions. The optimal surface IrO*_x_* composition achieves a balance between the binding of different OER intermediates with the best performance. We further use Au@Au_0.43_Ir_0.57_ as a bifunctional catalyst for overall water splitting. The voltage of the cell to reach a current density of 10 mA/cm^2^ is only 1.52 V, which outperforms the cell built by commercial couples Ir/C‖Pt/C (1.63 V). Remarkably, with only 0.02 mg_Ir_/cm^2^ loading, the overall water-splitting cell has a service life of at least 194 h, which is 1–2 orders of magnitude longer than the reported catalysts in acidic media. Given that Ir only exists on several layers (1–5) of the catalyst surface, this research is of great importance to the development of durable, highly efficient and cost-effective catalysts.

## RESULTS AND DISCUSSION

### Structural analysis of the catalysts

**Figure 1.**
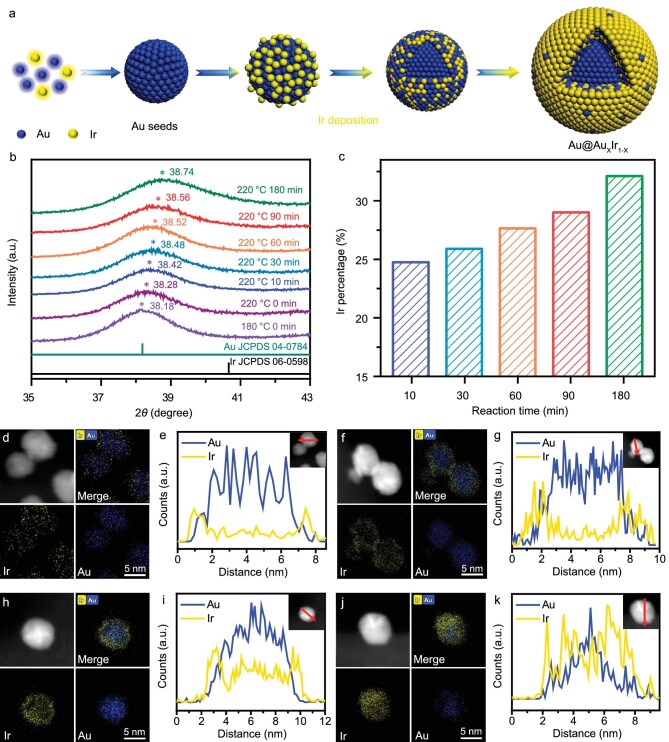
(a) Schematic illustration of the synthesis of Au@Au*_x_*Ir_1−_*_x_* core-shell NPs. (b) X-ray diffraction (XRD) patterns of Au@Au*_x_*Ir_1−_*_x_* core-shell NPs obtained at different intervals during the reaction (180°C, 0 min; 220°C, 0, 10, 30, 60, 90 and 180 min). (c) Relationship between the Ir percentage in whole NPs (estimated by ICP) and the reaction time. High-angle annular dark-field scanning TEM (HAADF-STEM) images and the corresponding energy dispersive X-ray spectroscopy (EDX) mappings of Au and Ir: (d) Au@Au_0.54_Ir_0.46_, (f) Au@Au_0.43_Ir_0.57_, (h) Au@Au_0.34_Ir_0.66_ and (j) Au@Au_0.26_Ir_0.74_. EDX line scan analyses of an NP along the arrows marked in the insets: (e) Au@Au_0.54_Ir_0.46_, (g) Au@Au_0.43_Ir_0.57_, (i) Au@Au_0.34_Ir_0.66_ and (k) Au@Au_0.26_Ir_0.74_.

The synthesis of Au@Au*_x_*Ir_1−_*_x_* core-shell NPs involves two stages: formation of the Au core and growth of the Au*_x_*Ir_1−_*_x_* shell through atomic diffusion (Fig. 1a). With an increase in reaction time, created by fixing the nominal ratio of precursors, the atomic ratio of Ir increased and the final product (Au@Au_0.34_Ir_0.66_) was obtained after a 3-h reaction (Fig. 1c, and inductively coupled plasma atomic emission spectrometry (ICP) and energy dispersive X-ray spectroscopy (EDS) results in Table S1). Au@Au_0.26_Ir_0.74_ was synthesized by increasing the amount of Ir precursor during a 3-h reaction. To monitor the formation process of the core-shell structure, a small number of NPs were taken out at different intervals during the reaction, and then the growth process was terminated by adding ethanol. Figure S1 shows the UV-vis spectra of bare Au and Au@Au*_x_*Ir_1−_*_x_* core-shell NPs obtained at different intervals during the reaction (180°C, 0 min; 220°C, 0, 10, 30, 60, 90 and 180 min). The absorption peak located at 524 nm is related to the formation of Au NPs (180°C, 0 min) [29]. This peak shifts to 515 nm at 220°C, indicating the deposition of Ir atoms on the Au seeds [30]. The peak shifts to 500 nm after a 30-minute reaction and does not have a significant shift even when extending the reaction time to 3 h. The X-ray diffraction (XRD) of Au@Au*_x_*Ir_1−_*_x_* NPs shows that all peaks are located between the characteristic peaks of Au and Ir, and will shift to the characteristic peaks of bulk Ir as the percentage of Ir increases (Fig. 1b and Fig. S2). These results suggest a potential growth process in which the Au nuclei are formed initially, and then the Au-Ir shell with different percentages of Ir is formed by an atomic diffusion process.

Transmission electron microscopy (TEM) and high-angle annular dark-field scanning TEM (HAADF-STEM) were used to characterize the morphologies and compositions of Au@Au*_x_*Ir_1−_*_x_* core-shell NPs (Fig. 1d–k and Fig. S3). For example, all NPs of Au@Au_0.43_Ir_0.57_ are uniformly loaded on carbon black with an average NP size of 7.83 ± 0.69 nm (Fig. S3b). The high-resolution TEM (HRTEM) image shows a lattice spacing of 2.25 Å in the shell and 2.34 Å in the core, corresponding to the (111) plane of Au_0.43_Ir_0.57_ and Au, respectively (Fig. S3b). The HAADF-STEM image of two random NPs and the corresponding energy dispersive X-ray spectroscopy (EDX) maps show the formation of a core-shell structure, in which Ir atoms mainly distribute in the shell region and Au is richer in the core part (Fig. 1f). The EDX line scan, which was taken along the red arrows and marked in the insets (Fig. 1g, and Figs S4b and S5), further suggests that the shell is composed of both Au and Ir with a thickness of ∼0.9 nm. All these results suggest the formation of an Au core and Au-Ir solid-solution shell. Other Au@Au*_x_*Ir_1−_*_x_* NPs all have an average particle size ranging from 7.50 nm to 7.98 nm (Fig. S3). HAADF-STEM images, the corresponding EDX maps, and line scan profiles show that all samples are core-shell structures (Fig. 1d–k and Fig. S4). The thickness of the Au*_x_*Ir_1−_*_x_* shell increases from 0.4 nm to 1.4 nm, with the average number of atomic layers tuned from 1 to 5 (Fig. S4). The average composition of Au : Ir in the shell was determined by EDS point analyses in 10 different locations, and the samples were named based on it (Fig. S6).

**Figure 2.**
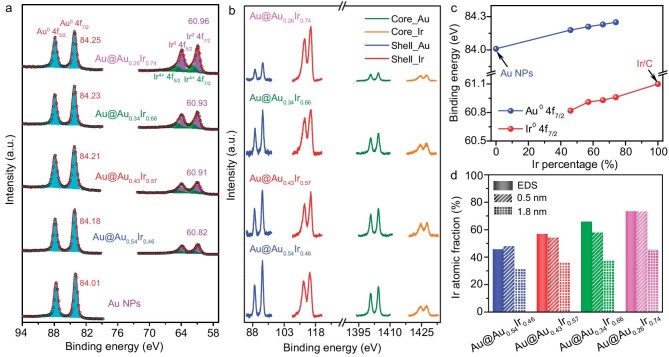
(a) X-ray photoelectron spectroscopy (XPS) spectra of Au@Au*_x_*Ir_1−_*_x_* and Au NPs. (b) Synchrotron radiation photoelectron spectroscopy (SRPES) spectra of Au@Au*_x_*Ir_1−_*_x_*. (c) A plot of the changes in the binding energy verses the metal composition of the shell. (d) Ir atomic fractions of as-synthesized Au@Au*_x_*Ir_1−_*_x_* NPs under different depths measured by SRPES and TEM-EDS.

To investigate the chemical states of Au@Au*_x_*Ir_1−_*_x_* NPs, XPS was performed. The Au 4f_7/2_ spectra of Au@Au*_x_*Ir_1−_*_x_* show a peak at 84.18–84.25 eV, which is ca. 0.2 eV higher than that of Au NPs (Fig. 2a) [31]. The Ir 4f spectra show two doublets of Ir^0^ 4f and Ir*^n^*^+^ (*n* > 0) 4f, corresponding to metallic Ir and IrO*_x_* species, respectively [32]. Compared to binding energies of Ir^0^ 4f_7/2_ in the Ir/C, Ir 4f spectra of Au@Au*_x_*Ir_1−_*_x_* are ca. 0.2–0.3 eV lower in the peak position (Fig. 2 and Fig. S7a). These results indicate that the electrons transfer from Au to Ir. With the Ir content increasing, the binding energy of Au^0^ 4f_7/2_ in Au-Ir linearly increases compared to Au NPs, and the peak shift of Ir^0^ 4f_7/2_ in Au-Ir relative to Ir/C decreases (Fig. 2c). Moreover, the electron-rich Ir atom is supposed to facilitate the formation of surface-active IrO*_x_* [33]. We further conducted density functional theory (DFT) calculations to acquire a surface charge analysis of Au@Au*_x_*Ir_1−_*_x_*. The Ir atoms on the surface of Au@Au*_x_*Ir_1−_*_x_* were negatively charged, which means it is easier to form the surface oxide layer with the trend to donate electrons (Table S2). The calculation results proved that the addition of Au can induce the generation of IrO*_x_*. Additionally, oxidation only occurs on the surface: if the Ir shell gets thickened, the surface oxidation ratio is lower. The percentage of IrO*_x_* in Au@Au*_x_*Ir_1−_*_x_* was semi-quantitatively analyzed with a sustained decrease from Au@Au_0.54_Ir_0.46_ to Au@Au_0.26_Ir_0.74_ as the Ir percentage increased (Fig. S7b). These results suggest that the fraction of IrO*_x_* can be controlled by tuning the composition of the Au*_x_*Ir_1−_*_x_* shell.

### In-depth X-ray photoelectron spectroscopy study

Synchrotron radiation photoelectron spectroscopy (SRPES) was further adopted to obtain the near-surface structural information of the catalyst [34]. Due to the adjustable photon energy, SRPES can characterize atomic composition information at different depths. SRPES spectra of Au@Au*_x_*Ir_1−_*_x_* were measured at photon energies of 180 and 1486 eV for Au 4f and Ir 4f under ultrahigh vacuum (UHV) using Al *K*α X-rays source (Fig. 2b). The mean free paths of Au 4f and Ir 4f photoelectrons generated at 180 and 1486 eV were ∼0.5 nm and 1.8 nm [35], which can be correlated to the near-surface (shell) and inside of the particle (core) regions. All Au@Au*_x_*Ir_1−_*_x_* NPs have a similar core-shell structure with an Au-rich core but there are obvious differences in the composition of the shells. The shell of Au@Au_0.54_Ir_0.46_ is Au-rich, and with Ir content increasing, Au@Au_0.26_Ir_0.74_ exhibits an Ir-rich shell. The Ir atomic fraction of as-synthesized Au@Au*_x_*Ir_1−_*_x_* NPs under different depths (0.5 nm and 1.8 nm) is shown in Fig. 2d. The observed atomic fraction of these NPs is 0.48 (Au@Au_0.54_Ir_0.46_), 0.54 (Au@Au_0.43_Ir_0.57_), 0.58 (Au@Au_0.34_Ir_0.66_) and 0.74 (Au@Au_0.26_Ir_0.74_) within a mean free path distance of 0.5 nm, which is consistent with the EDS results in the near-surface areas (see details in the supporting information).

### Electronic structure of iridium centers

**Figure 3.**
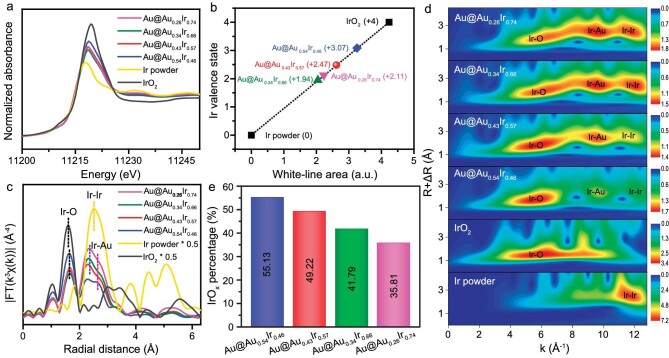
(a) Ir *L*_3_-edge X-ray absorption near-edge structure (XANES) spectra. (b) The valence states of Ir from ΔXANES spectra. (c) *k*^3^-weighted Fourier-transform *L*_3_-edge extended XAFS (EXAFS) spectra and (d) wavelet transform for the *k*^3^-weighted Ir *L*_3_-edge EXAFS signal for Au@Au*_x_*Ir_1−_*_x_* and standard samples. (e) IrO*_x_* percentage (estimated by EXAFS analysis) for Au@Au*_x_*Ir_1−_*_x_*.

The valence state and local coordinate structure of Au@Au*_x_*Ir_1−_*_x_* were further characterized by XAFS. Owing to the fully filled 5*d* orbital of Au, there is almost no difference in the X-ray absorption near-edge structure (XANES) and Fourier-transform extended XAFS (FT-EXAFS) of the Au *L*_3_-edge between Au@Au*_x_*Ir_1−_*_x_* and Au foil (Fig. S8). This indicates that the coordination environment of Au in the Au@Au*_x_*Ir_1−_*_x_* is similar. The XANES spectra of the Ir *L*_3_-edge of Au@Au*_x_*Ir_1−_*_x_* have similar oscillations to that of Ir powder, which indicates the similar atomic configuration of the Ir site in both Au@Au*_x_*Ir_1−_*_x_* and metallic Ir (Fig. 3a). The order of white-line intensity is IrO_2_ > Au@Au_0.54_Ir_0.46_ > Au@Au_0.43_Ir_0.57_ > Au@Au_0.26_Ir_0.74_ > Au@Au_0.34_Ir_0.66_ > Ir powder, which suggests the existence of coordinated unsaturated Ir sites in Au@Au*_x_*Ir_1−_*_x_* [36]. Figure S9 shows the differential XANES (ΔXANES) spectra for Ir *L*_3_-edge XANES, normalized by using Ir powder as the reference [37]. The Ir valence state was confirmed by integration of the white-line peak area in the ΔXANES spectra (Fig. 3b), which is 3.07, 2.47, 1.94 and 2.11 for Au@Au_0.54_Ir_0.46_, Au@Au_0.43_Ir_0.57_, Au@Au_0.34_Ir_0.66_ and Au@Au_0.26_Ir_0.74_, respectively. Thus, the change in the electronic structure of Ir with varied compositions is due to the homogenously mixed Au and Ir atoms on an atomic scale.

In the FT-EXAFS spectra of Ir *L*_3_-edge, compared with standard Ir powder and IrO_2_, two peaks at ca. 1.60 Å and 2.40 Å belong to Ir–O and Ir–Ir coordination, respectively (Fig. 3c) [38]. However, due to the similar bond length between Au and Ir, the FT-EXAFS spectra of Au@Au*_x_*Ir_1−_*_x_* deliver the main peak located at 2.40 Å with a shoulder peak at ca. 2.60 Å, and it is difficult to directly determine the coordination patterns in the materials through the FT-EXAFS spectra. Thus, wavelet transform (WT) EXAFS was used to directly confirm the existence of Ir sites in different coordination environments by a combination of *R* space with *K* space (Fig. 3d) [39]. IrO_2_ and Au@Au*_x_*Ir_1−_*_x_* have an intensity maximum near 6 Å^−1^, due to the contribution of the Ir–O. Apart from the intensity maximum at *k* = 11.7 Å^−1^ belonging to Ir–Ir, all Au@Au*_x_*Ir_1−_*_x_* samples show another intensity maximum at *k* = 9.5 Å^−1^ because of Ir–Au coordination. The first shell quantitative fitting on the EXAFS curve suggests that the scattering at ca. 1.60, 2.40 and 2.60 Å is attributed to the coordination of Ir–O, Ir–Ir and Ir–Au, respectively (Figs S10, S11 and Table S3). This result indicates the interaction between Ir and Au, as well as the presence of metals and metal oxides in the sample. As the proportion of Ir is increased, the coordination number of Ir–Au and Ir–O decreases, while the coordination number of Ir–Ir increases (Fig. S12). The IrO*_x_* percentage decreases from 55.13% (Au@Au_0.54_Ir_0.46_) to 35.81% (Au@Au_0.26_Ir_0.74_). The XAFS results are consistent with the XPS analyses, which confirm that the composition of the shell can regulate the electronic structure of Ir and the formation of IrO*_x_* (Fig. 3e).

### Comparisons of OER performance

**Figure 4.**
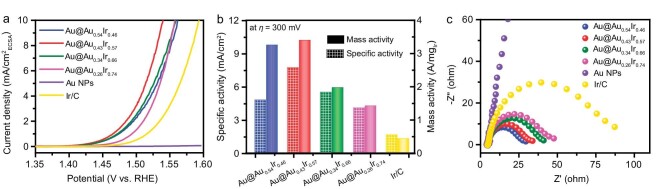
(a) OER polarization curves normalized by electrochemically active surface area (ECSA). (b) Specific and mass activities at *η* = 300 mV. (c) Electrochemical impedance spectroscopy (EIS) measurements of Au@Au*_x_*Ir_1−_*_x_* and commercial Ir/C, at 1.52 V and with a frequency range of 100 kHz–10 mHz. All LSV data are given with 90% *i*R-compensation.

The OER activity of Au@Au*_x_*Ir_1−_*_x_* catalysts was evaluated by linear sweep voltammetry (LSV) along with commercial Ir/C and IrO_2_ as references and with the loading of 10.2 *μ*g_Ir_/cm^2^ for all catalysts (Fig. S13a and Table S4). The overpotentials reaching 10 mA/cm_geo_^2^ (geo: geometric area of the electrode, 0.196 cm^2^) of Au@Au*_x_*Ir_1−_*_x_* (Au_0.54_Ir_0.46_, Au_0.43_Ir_0.57_, Au_0.34_Ir_0.66_ and Au_0.26_Ir_0.74_) are 255, 257, 273 and 289 mV, respectively, which are much lower than those of Ir/C (325 mV) and IrO_2_ (364 mV) (Fig. S14a), and surpass most of the Ir-based catalysts reported (Table S5). To evaluate the intrinsic activities, we compared the specific activity (Fig. 4a) by normalizing the current densities to the electrochemically active surface area (ECSA), which was measured by calculating the hydrogen underpotential deposition area from the cyclic voltammetry curves (Figs S15–S18). Since the applied upper potential was far more negative than the onset potential of Au (ca. 2.0 V), only the quality of Ir was considered when ECSA was calculated. Additionally, we also compared the mass activity due to its importance in the practice device design. All the Au@Au*_x_*Ir_1−_*_x_* catalysts have higher specific and mass activities than the commercial Ir/C, so the high dispersion of Ir atoms in the core-shell structure leads to enhanced activity.

The electronic interaction between Au and Ir can cause partial oxidation of Ir and form surface IrO*_x_*, which provides a balance for different intermediates binding, and thus enhances OER performance [24]. However, the influence of the IrO*_x_* amount on the catalytic properties is still unknown. The above results confirm that the surface fraction of IrO*_x_* was successfully controlled by tuning the Au/Ir ratio, which allows us to correlate the OER activity with the faction of IrO*_x_*. As shown in Fig. 4b, at *η* = 300 mV, both specific and mass activities of Au@Au*_x_*Ir_1−_*_x_* catalysts display a volcano-shaped relationship with the atom ratio of the shell (or fraction of IrO*_x_*). Au@Au_0.43_Ir_0.57_ with a medium IrO*_x_* fraction (49.22%) locates at the vertex (7.77 mA/cm^2^ and 3.40 A/mg) showing 4.5- and 7.7-times higher activities than the commercial Ir/C (1.71 mA/cm^2^ and 0.44 A/mg). We further assessed charge transfer capability by electrochemical impedance spectroscopy (EIS) measured at an overpotential of 290 mV. Equivalent circuit fitting of EIS data was performed to quantitatively evaluate their charge transfer capability (Fig. S19). Au@Au_0.43_Ir_0.57_ and Au@Au_0.54_Ir_0.46_ with a higher fraction of IrO*_x_* have lower reaction resistance than other Au@Au*_x_*Ir_1−_*_x_*, Ir/C and Au NPs, indicating that highly dispersed IrO*_x_* active sites accelerated the charge transfer process (Fig. 4c and Table S6). Although experimental observation of the crystal structure of amorphous IrO*_x_* remains a challenge now, our results suggest the importance of controlling the surface IrO*_x_* fraction for OER in the Ir-based nanocatalysts. Moreover, the OER durability of Au@Au_0.43_Ir_0.57_ was conducted by chronopotentiometric test at 10 and 100 mA/cm^2^ (Fig. S20). The potential of Au@Au_0.43_Ir_0.57_ was almost constant for at least 40 h, representing a rarely reported stable OER activity in such a strong acid. The good durability comes from the strong electronic interaction between Au and Ir as well as the intrinsic stability of Au under an OER condition (Fig. S21) [5,24].

### DFT calculations of the electronic structure and valence band photoemission

**Figure 5.**
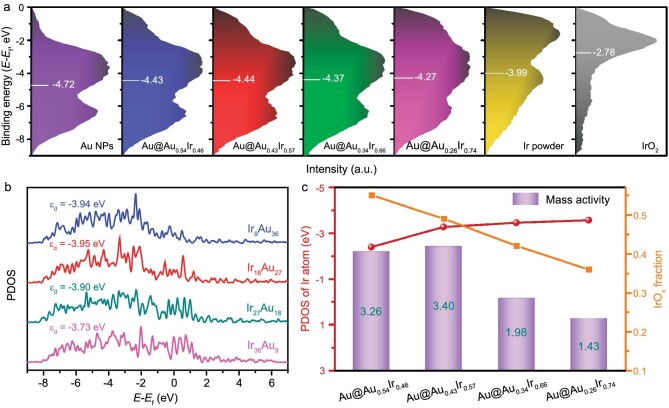
(a) Valence band photoemission spectra of Au@Au*_x_*Ir_1−_*_x_* and standard samples. The white bar represents the *d*-band center. All the spectra were background-corrected. (b) DFT calculated PDOS of *d*-bands and *d*-band centers for Au-Ir systems. (c) Structure–activity relationships.

In the typical four proton-coupled electron transfer OER reaction, the binding energies of *O, *OH and *OOH are not independent but interrelated [40]. Given the linear correlation between the binding energies of *OOH and *OH, the scaling equation (ΔG_*OOH_ = ΔG_*OH_ + 3.2 eV) shows [41] that the activity is related to oxygen binding energy. The representative *d*-band center has been proposed as a descriptor for finding optimal catalysts [42,43]. The electronic structure of the catalyst can affect the binding strength of the intermediate, and the binding strength is determined by the interaction between the adsorbate and the *d*-band for the transition metal [44–46]. Moreover, owing to the occupancy of anti-bonding states, the trend is that the higher position of the *d*-band relative to the Fermi level leads to stronger binding [47]. The electronic structures of Au@Au*_x_*Ir_1−_*_x_* and standard samples were analyzed by the valence band photoemission, and the quantitative information of the *d*-band center was obtained through calculation. As shown in Fig. 5a and Table S7, the position of the *d*-band center for Ir powder is −3.99 eV, and when introducing Au (−4.72 eV), the positions were shifted to −4.27 eV (Au@Au_0.26_Ir_0.74_), −4.37 eV (Au@Au_0.34_Ir_0.66_), −4.44 eV (Au@Au_0.43_Ir_0.57_) and −4.43 eV (Au@Au_0.54_Ir_0.46_), which are much lower than the Fermi level. In addition, compared with metallic Ir, rutile Ir oxides displayed a higher *d*-band center (−2.78 eV) and led to the declined OER activity. The IrO*_x_* species on the Au@Au*_x_*Ir_1−_*_x_* surface is generally considered the active species for OER. However, too much IrO*_x_* will lead the *d*-band center of the catalyst close to the Fermi level, causing a too-strong binding affinity of intermediates that needs a quite high overpotential for the OER. Therefore, an optimal fraction of IrO*_x_* combined with a suitable *d*-band center may result in the best OER activity.

To reveal the intrinsic electronic effect of the Au-Ir core-shell systems with different ratios between Au and Ir on the performance of water splitting, DFT calculations were further conducted to acquire the partial density of states (PDOS) for different Au-Ir systems (Figs S22 and S23). As shown in Fig. 5b and Fig. S24, the positions of *d*-bands for Au*_x_*Ir*_y_* are shifting away from the Fermi level with the increase in the ratio of Au in the Au-Ir systems, resulting in a decreased tendency of *d*-band center for Au_*x*_Ir_*y*_. The calculated *d*-band centers (*ε*_d_) follow the trend: Ir_36_Au_9_ (−3.73 eV) > Ir_27_Au_18_ (−3.90 eV) > Ir_9_Au_36_ (−3.94 eV) > Ir_18_Au_27_ (−3.95 eV), correlating with the valence band photoemission result. Ir is the active site, so we focused on the PDOS of Ir atoms on the Au_*x*_Ir_*y*_ surface. Figure S24 and Table S8 show the calculated ε_d_ order for Ir atoms: Ir_36_Au_9_ (−3.57 eV) < Ir_27_Au_18_ (−3.46 eV) < Ir_18_Au_27_ (−3.27 eV) < Ir_9_Au_36_ (−2.40 eV), as an inverse relationship with the ratio of Ir. Thus, the strong binding between Ir active sites and adsorbed oxygenated species occurred in Au-Ir systems, and the enhanced Ir–O binding strength via the introduction of Au boosted the intrinsic OER activity [48]. However, a too-strong or too-weak combination of intermediates will not be conducive to the progress of the reaction, so too high or too low position of *d*-band will reduce the electrocatalytic activity [49]. Combined with the experimental result that Au@Au_0.43_Ir_0.57_ exhibited the best intrinsic activity toward OER, our calculations revealed that a suitable Ir *d*-band center combined with the optimal fraction of IrO*_x_* can achieve a better balance for different oxygenated intermediates binding, and thus improve the OER activity (Fig. 5c and Fig. S25).

### HER and overall water-splitting performance

**Figure 6.**
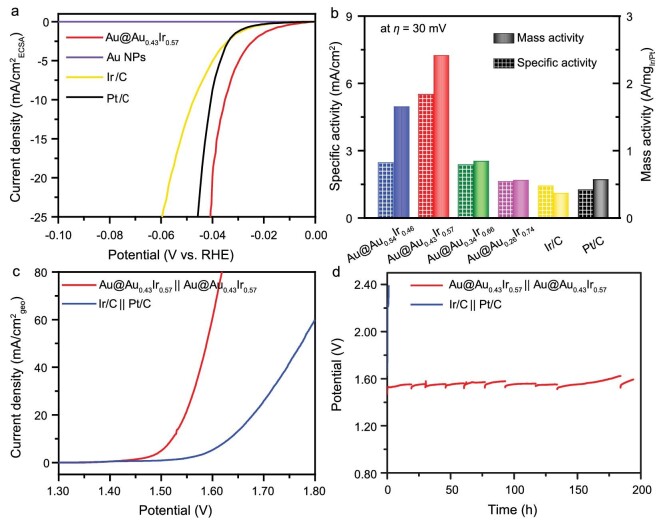
(a) HER polarization curves of Au@Au_0.43_Ir_0.57_, Au NPs, commercial Ir/C and Pt/C in 0.5 M H_2_SO_4_ solution, normalized by ECSA. (b) Specific and mass activities of Au@Au*_x_*Ir_1−_*_x_* at *η* = 30 mV relative to commercial Ir/C and Pt/C catalysts. (c) Polarization curves for overall water splitting. (d) Chronopotentiometric test at 10 mA/cm^2^ for overall water splitting of Au@Au_0.43_Ir_0.57_‖Au@Au_0.43_Ir_0.57_ and Ir/C‖Pt/C.

Besides the superior OER performance, Au@Au_0.43_I_r0.57_ shows good HER performance (Fig. 6a and Fig. S26) with a lowest overpotential of only 23 mV at 10 mA/cm^2^ and Tafel slope (17.05 mV/dec), which is much lower than that of Pt/C (33 mV, 15.67 mV/dec) and Ir/C (38 mV, 17.82 mV/dec) (Figs S13b, S14b and S27b). To our knowledge, Au@Au_0.43_Ir_0.57_ is superior to most HER catalysts in acidic media (Table S9). Importantly, the specific and mass activities of Au@Au_0.43_Ir_0.57_ at *η* = 30 mV are 5.51 mA/cm^2^ and 2.41 A/mg, which are 4.4 and 4.2 times those (1.25 mA/cm^2^ and 0.57 A/mg) of commercial Pt/C, respectively (Fig. 6b).

Encouraged by the excellent HER and OER performances of Au@Au_0.43_Ir_0.57_, we further tested the overall water-splitting activity by using it as both anode and cathode in a two-electrode home-made cell configuration [50]. The cell voltage, reaching 10 mA/cm^2^ for Au@Au_0.43_Ir_0.57_‖Au@Au_0.43_Ir_0.57_, is only 1.52 V, which is 110 mV lower than that of commercial Ir/C‖Pt/C (1.63 V) and outperforms the recently reported Ir-based catalysts in acidic electrolyte (Fig. 6c and Table S10). Importantly, with only 0.02 mg_Ir_/cm^2^ loading, the Au@Au_0.43_Ir_0.57_‖Au@Au_0.43_Ir_0.57_ went for more than 194 h at a cell current density of 10 mA/cm^2^, which is much more durable than the Ir/C‖Pt/C (11 min) (Fig. 6d). Specifically, after 184 h and 194 h of chronopotentiometric testing, *η* at 10 mA/cm_geo_^2^ of Au@Au_0.43_Ir_0.57_ were only slightly increased at 11 and 13 mV, respectively (Fig. S28). What is more, it is stable for at least 320 h at a high current density of 100 mA/cm^2^ (Fig. S29). We characterized the samples after stability testing by inductively coupled plasma optical emission spectroscopy (ICP-OES) (Table S11) and TEM (Fig. S30). The results showed that only a small amount of Ir was dissolved, and the core-shell morphology was still maintained. The reason for achieving long-term stability is that the design of the highly efficient catalyst significantly reduces the reaction overpotential so that the working potential range is lower than the metal dissolution potential. This performance represents the best durability among the reported bifunctional electrocatalysts in acidic media (Table S12).

## CONCLUSIONS

In summary, we first synthesized a range of Au@Au*_x_*Ir_1−_*_x_* core-shell nanocatalysts composed of an Au core and Au*_x_*Ir_1−_*_x_* alloy shell. The Au/Ir ratio in the shell was controlled from 1 : 0 to 0.26 : 0.74 following an atomic diffusion process. SRPES and XAFS results revealed the gradually changing fraction of surface IrO*_x_* with a change in Au/Ir compositions, which confirms the correlation between water-splitting performance and IrO*_x_* percentage. Among all kinds of Au@Au*_x_*Ir_1−_*_x_*, Au@Au_0.43_Ir_0.57_ with a medium fraction of IrO*_x_* exhibits an optimal performance for both OER and HER. For OER, the specific and mass activities of Au@Au_0.43_Ir_0.57_ at *η* = 300 mV are 4.5 and 7.7 times those of commercial Ir/C. For HER, the specific and mass activities of Au@Au_0.43_Ir_0.57_ at *η* = 30 mV are 4.4 and 4.2 times those of commercial Pt/C, respectively. EIS results suggest that a suitable amount of IrO*_x_* accelerates OER charge transfer kinetics, with a balance binding of the OER intermediates. With only a 0.02 mg_Ir_/cm^2^ loading amount, the voltage of the water-splitting cell built by Au@Au_0.43_Ir_0.57_ to reach a current density of 10 mA/cm^2^ is only 1.52 V, with a service life of at least 194 h, which is the best durability in acidic media. This work offers an example of increased mass activity without sacrificing durability, and provides practical applications for the development of PEMWEs.

## METHODS

### Reagents and chemicals

Hydrogen tetrachloroaurate (III) trihydrate (HAuCl_4_·3H_2_O, 99.9%) and 5 wt% Nafion solution (∼5% in lower aliphatic alcohols and water, contains 15%–20% water) were purchased from Sigma-Aldrich. Iridium (III) chloride hydrate (IrCl_3_·*x*H_2_O, 99.9%) and Iridium (IV) oxide powder (IrO_2_, 99%) were purchased from Alfa Aesar. Oleylamine (80%–90%) was purchased from Acros. Carbon black (Vulcan XC-72), commercial Pt/C (20% Pt on Vulcan XC-72) and commercial Ir/C (20% Ir on Vulcan XC-72) were purchased from Premetek. Cyclohexane (C_6_H_12_, A.R.), hexane (C_6_H_14_, A.R.), ethanol (C_2_H_5_OH, A.R.) and iso-propyl alcohol (C_3_H_8_O, A.R.) were purchased from Sinopharm Chemical Reagent Co. Ltd. All the chemicals were used as received without further purification. The water used in all experiments was ultrapure (Millipore, 18.25 MΩ/cm).

### Synthesis of Au@Au*_x_*Ir_1−_*_x_* core-shell nanoparticles

Synthesis of Au@Au_0.43_Ir_0.57_ core-shell NPs: In a typical synthesis of Au@Au_0.43_Ir_0.57_, 0.1334 mmol HAuCl_4_·3H_2_O and 0.0667 mmol IrCl_3_·*x*H_2_O were dissolved in 10 mL oleylamine by ultrasonication for several minutes. Then, the mixture was put into a preheated metal bath, and heated at 220°C for 1.5 h under a nitrogen atmosphere. After the flask was cooled to room temperature, the brown products were centrifuged with ethanol at 10 000 r/min for 5 min and then washed with a mixture of ethanol/cyclohexane eight times. Finally, the as-synthesized Au@Au_0.43_Ir_0.57_ was redispersed in cyclohexane for reserve.

Synthesis of Au@Au*_x_*Ir_1−_*_x_* core-shell NPs: The synthesis of Au@Au*_x_*Ir_1−_*_x_* core-shell NPs with different shell thicknesses was similar to that of Au@Au_0.43_Ir_0.57_ except that the reaction time was adjusted from 90 min to 0, 10, 30, 60 and 180 min. These samples are named correspondingly: AuIr_0.22_, Au@Au_0.54_Ir_0.46_, Au@Au_0.48_Ir_0.52_, Au@Au_0.45_Ir_0.55_ and Au@Au_0.34_Ir_0.66_.

Synthesis of Au@Au_0.26_Ir_0.74_ core-shell NPs: The synthesis of Au@Au_0.26_Ir_0.74_ core-shell NPs was similar to that of Au@Au_0.43_Ir_0.57_ except that the amount of IrCl_3_·*x*H_2_O was increased from 0.0667 mmol to 0.0800 mmol.

## Supplementary Material

nwae056_Supplemental_File
