# Supplementary material for: Sub-10-nm-sized Au@AuxIr1−x metal-core/alloy-shell nanoparticles as highly durable catalysts for acidic water splitting
Source: Natl Sci Rev. 2024 Feb 7;11(4):nwae056. doi: 10.1093/nsr/nwae056 (PMC10914371; doi:10.1093/nsr/nwae056)
Supplement: nwae056_Supplemental_File [file nwae056_supplemental_file.pdf]

Supporting Information

Sub-10 nm sized $Au@Au_xIr_{1-x}$ Metal-Core/Alloy-Shell Nanoparticles as highly durable catalysts for Acidic Water Splitting

Huimin Wang[1,4], Zhe-ning Chen[1], Yuanyuan Wang[2], Dongshuang Wu[2*], Minna Cao[1,4*], Fanfei Sun[3], Rong Cao[1,4,5 *]

[1] State Key Laboratory of Structural Chemistry, Fujian Institute of Research on the Structure of Matter, Chinese Academy of Sciences, Fuzhou, 350002, P. R. China.

[2] School of Materials Science and Engineering, Nanyang Technological University, 639798, Singapore.

[3] Shanghai Synchrotron Radiation Facility, Shanghai Institute of Applied Physics, Chinese Academy of Sciences, Shanghai, 201204, P. R. China.

[4] University of Chinese Academy of Sciences, Beijing, 100049, P. R. China.

[5] Fujian Science & Technology Innovation Laboratory for Optoelectronic Information of China, Fuzhou, 350108, P. R. China.

Table of contents

1. Syntheses 3

2. Characterizations and Electrochemical Measurements 3

3. Calculation details 7

4. Supplementary Figures and Tables 9

5. References 46

1. Syntheses

1.1 Synthesis of Au nanoparticles. In a typical synthesis of Au nanoparticles, 0.2 mmol $HAuCl_4 \cdot 3H_2O$ was dissolved in 15 mL oleylamine by ultrasonication for several minutes. Then, the mixture was put into a preheated metal bath and was heated to 180 ℃. The reaction was kept at 180 ℃ for 30 min under a nitrogen atmosphere and then was allowed to cool down to room temperature. The purple product was centrifuged by adding ethanol and centrifuged at 10000 rpm for 5 min, and following by a washing process using a mixture of ethanol/cyclohexane 8 times. Finally, as-synthesized Au nanoparticles were redispersed in cyclohexane for reserve.

1.2 Synthesis of C-supported Au@Au_xIr_{1-x} catalysts. To prepare our Au@Au_xIr_{1-x} electrocatalysts, we mixed and stirred a hexane dispersion of Au@Au_xIr_{1-x} with 70 mg Carbon black (Vulcan XC-72) for 2 h, after which hexane was evaporated from the solution. The catalysts were washed with ethanol and dried under a vacuum.

2. Characterizations and Electrochemical Measurements

2.1 Instrumentation. Transmission electron microscopy (TEM), high-resolution transmission electron microscopy (HRTEM), energy-dispersive X-ray spectroscopy analysis (EDS), scanning transmission electron microscopy (STEM), and STEM-EDS element mapping were conducted on Talos-F200X scanning/transmission electron microscope (S/TEM) (Thermo Scientific, America) respectively operated at 200 kV. Inductively coupled plasma atomic emission spectrometry (ICP) was performed with Ultima2 inductively coupled plasma OES spectrometer (ICP-OES, Agilent 730) to analyze the elemental composition of the materials. Powder X-ray diffraction (PXRD) analysis was carried out on Miniflex-600 X-ray diffractometer (Rigaku, Japan), with scanning speed of 0.2 °/min under Cu *K*α radiation (λ =1.5406 Å). UV-visible absorption spectra (UV-vis) were recorded on a UV–vis spectrophotometer (Shimadzu, UV–2550). X-ray photoelectron spectroscopy (XPS) was conducted on X-ray photoelectron spectrometer (ESCALAB 250Xi, Thermo Fisher) with a monochromatic Al *K*α (1486.7 eV) X-ray source and a chamber pressure of 5×10^{-10} mbar.

2.2 XAFS measurements. The X-ray absorption fine structure spectra measurements (Au L_3-edge

and Ir L_3-edge) were investigated by the BL14W1 in Shanghai Synchrotron Radiation Facility (SSRF). The electron beam energy was 3.5 GeV and the stored current was 230 mA (top-up). XAFS data were collected using a fixed-exit double-crystal Si (111) monochromator. A Lytle detector was used to collect the fluorescence signal, and the energy was calibrated using metals foil. All samples were pelletized into disks of 5 mm diameter with 1mm thickness. The obtained EXAFS data was performed utilizing the Athena module in the IFEFFIT package, following standard procedures. Based on the pre-processed data, the pre-edge and post-edge backgrounds were deducted from the overall absorption and then normalized to obtain the EXAFS spectra. The EXAFS contributions were separate from different coordination shells by Fourier transforming the χ(k) function to real (R) space using a hanging windows (dk=1.0 $Å^{-1}$). Subsequently, the ARTEMIS module in the IFEFFIT package was used to the least-squares curve parameter fitting to obtain the quantitative structural parameters around Au and Ir atoms. During the curve-fitting of Ir L_3-edge, the overall amplitude reduction factor $S0_2$ was fixed to the best-fit value determined from fitting the data of metal Ir powder. For the Au@Au_xIr_{1-x}, the structural parameters, including the coordination number N, interatomic distance R, the Debye-Waller factor σ^2 and the edge-energy shift E_0 were allowed to vary during the fitting process. The bond distances were adjusted based on initial inputs from standard crystal structure information files of Ir and Au for the fits of Au-Au bond and Au-Ir bond, respectively.

2.3 Synchrotron radiation photoelectron spectroscopy (SRPES). Synchrotron radiation photoelectron spectroscopy was performed at the Photoemission Endstation (BL10B) in the National Synchrotron Radiation Laboratory (NSRL), China. The end station of the BL10B beamline is a combined ultra-high vacuum (UHV) system including the analysis chamber, the preparation chamber, and a radial distribution chamber. The base pressures are 7×10^{-11} mbar, 2×10^{-10} mbar, and 7×10^{-11} mbar, respectively. The analysis chamber is equipped with a VG Scienta R4000 analyzer, a monochromatic Al *K*α X-ray source, a UV light source, low energy electron diffraction (LEED), and a flood electron gun. The structure of Au@Au_xIr_{1-x} was measured at photon energies of 180 and 1486 eV for Au 4f and Ir 4f, under ultrahigh vacuum (UHV) using Al *K*α x-rays source.

The Ir atomic fractions as-synthesized Au@Au_xIr_{1-x} NPs was determined by SRPES, and the

number of gold or iridium atoms within the same detection region is proportional to the peak areas. The Au 4f or Ir 4f peak areas were calibrated by the incident photo flux and atomic ionization cross-sections under the corresponding X-ray energies [1], and then divide the number of Ir atoms by the total number of metal atoms to obtain the Ir atomic fraction. The Ir atomic fraction was calculated as follow:

$$AF_{Ir} = \frac{S_{Ir}/(Flux * AICS_{Ir})}{N_{metal}}$$

$$N_{metal} = \frac{S_{Ir}}{Flux * AICS_{Ir}} + \frac{S_{Au}}{Flux * AICS_{Au}}$$

AF_{Ir}: Ir atomic fraction;

S: the fitting peak area;

Flux: the incident photo flux;

AICS: atomic ionization cross section;

N_{metal}: the total number of metal atoms.

2.4 Electrochemical Measurements.

Preparation of the working electrode. 4.0 mg of the as-synthesized $Au@Au_xIr_{1-x}$ *catalysts* was dispersed in a mixture of 1940 μL isopropanol and 60 μL Nafion (5 wt % Nafion, Sigma-Aldrich) by ultrasonic treating for about 2 h. According to ICP-OES, appropriate volume of the catalyst ink was pipetted onto a pre-cleaned glassy carbon rotating disk electrode (RDE) with a geometric area of 0.196 cm^2, leading to the metal Ir loading of 10.2 μg /cm^2, and then the as-prepared WE was dried at room temperature. A commercial Pt/C and Ir/C electrocatalysts (20 wt % metal on Vulcan XC-72 carbon support, Premetek Co.) were applied as a reference for benchmarking purpose. Similar to the carbon-supported $Au@Au_xIr_{1-x}$ catalysts, 4.0 mg of the commercial Ir/C or Pt/C was dispersed in a mixture of 1940 μL isopropanol and 60 μL Nafion under ultrasonication for 2 h. 5 μL of the suspension was deposited on a clean glassy carbon

RDE and dried at room temperature. The loading amounts of Pt or Ir for the commercial electrocatalysts on RDE were also 10.2 $\mu g/cm^2$.

Electrochemical measurements. HER and OER electrochemical measurements were measured by an IM6 electrochemical workstation (Zahner, Germany) equipped with a rotating disk electrode (RDE) (PINE 710, USA) in a three-electrode electrolytic cell. In this system, a Platinum net electrode (for OER, 1 cm^2), carbon rod electrode (for HER) and Ag/AgCl (Gaoss Union, saturated KCl) were used as counter electrode and reference electrode, respectively. Cyclic voltammetric (CV) curves were collected at a scan rate of 100 mV/s at room temperature in N_2-saturated 0.5 M H_2SO_4 solution. Before electrocatalysis measurements, the cyclic voltammetry was used to remove covering surfactants from catalysts in 0.5 M H_2SO_4 at a sweep rate of 100 mV/s from 0.2 to 0.6 V. The OER electrocatalytic activity was performed in O_2-saturated 0.5 M H_2SO_4 solution using linear sweep voltammetry from 1.0 V to 1.4 V (versus Ag/AgCl) with the scan rate of 10 mV/s. The HER electrocatalytic activity was tested in N_2-saturated 0.5 M H_2SO_4 solution using linear sweep voltammetry from -0.1 V to -0.3 V (versus Ag/AgCl) with the scan rate of 10 mV/s. All HER and OER measurements were conducted under rotating at 1600 rpm to remove the produced bubbles at room temperature, with 90% *i*R-drop correction. The potentials reported in this article were all calibrated and converted to the RHE using the equation

$E_{RHE} = E_{Ag/AgCl} + 0.198\ V + 0.0591 \times pH$.

Overall water splitting measurements. The overall water splitting was investigated in a two-electrode system in 0.5 M H_2SO_4. The catalyst was directly loaded on carbon fiber papers (CFP) without Carbon black (0.5 cm×1.5 cm, effective loading area was 0.5 cm×0.5 cm) and used as both anode and cathode (loading amount of 20.0 $\mu g_{Ir}/cm^2$). The device was labelled as $Au@Au_{0.43}Ir_{0.57}||Au@Au_{0.43}Ir_{0.57}$. For comparison, Ir/C and Pt/C were employed as the anode and cathode catalysts in the same configuration, which was labeled as Ir/C||Pt/C. The chronopotentiometry test of overall water splitting was operated at a constant current density of 10 mA/cm^2 for 194 h.

Electrochemically active surface area (ECSA). The initial specific electrochemically active surface areas (ECSAs) of Pt/C, Ir/C, and $Au@Au_xIr_{1-x}$ catalysts were measured by calculating

the hydrogen underpotential deposition area (H_{upd}) from the cyclic voltammetry curves [2]. These values were evaluated from the third cycle of a total of five CV scans performed between +0.05 and +1.05 V vs RHE for Pt/C and +0.05 and +0.55 V vs RHE for Ir-based catalysts, with a 100 mV/s scan rate in N_2-saturated 0.5 M H_2SO_4 solution. The ECSA of each electrocatalyst was calculated based on charges associated with the desorption of hydrogen in the region of 0.06–0.4 V after double-layer correction with a reference value of 210 $\mu C/cm^2$. The ECSA of Au was estimated from the reduction peak charge of Au oxides (390 $\mu C/cm^2$), measured from CV between 0.2 and 1.7 V at a sweep rate of 100 mV/s in N_2-saturated 0.5 M H_2SO_4 solution.

Electrochemical impedance spectroscopy (EIS). The EIS was measured in the range of 100k-10m Hz with an amplitude of 5 mV. For OER, the external voltage was 1.52 V (at the overpotential of 290 mV) vs RHE. ZSimpWin was used for equivalent circuit fitting of EIS data. The fitting data is shown in table S4.

2.5 Valence band photoemission spectra. The *d*-band center was determined from valence band photoemission spectra. All spectra have been baseline-corrected using a Shirley background. The binding energies were given with relative to the Au Fermi edge (E_F), assuming that the E_F is at 0 eV. The position of the *d*-band center was calculated through the integration from 0 to -9.0 eV. The *d*-band center position was calculated as follow:

$$d = \frac{\int N(\varepsilon)\varepsilon d\varepsilon}{\int N(\varepsilon) d\varepsilon}$$

$N(\varepsilon)$: the density of state (the photoelectron intensity after background subtraction) [3,4].

3. Calculation details

3.1 DFT Calculations.

Computational Models and Method. Au-Ir core-shell system was constructed based on the Ir(111) surface model, in which the component gradually changes from the pure Au on the bottom layer, to the pure Ir on the top layer. During structural optimization, the bottom two layers were frozen and the top three layers and adsorbed species were fully relaxed. All the electronic structure calculations were performed using the plane-wave periodic density functional theory as implemented in the Vienna *ab initio* simulation package (VASP) [5-7]. The projector

augmented-wave (PAW) method developed by Blöchl [8] to describe the electron–ion interactions using plane wave basis sets was employed. The generalized gradient approximation (GGA) with the Perdew-Burke-Ernzerh (PBE) exchange-correlation functional was used [9]. The kinetic energy cutoff was set to 450 eV. The convergence criteria for the energy calculations were set to a self-consistent field (SCF) tolerance of 1.0×10^{-5}eV. All internal structure parameters were relaxed until the maximum Hellmann−Feynman force on each ion were less than 0.02 eV/Å. Integration over the Brillouin zone was achieved with k-points mesh of 5*5*1 according to the Monkhorst-Pak scheme [10] together with a smearing method in Methfessel-Paxton scheme (order 1) broadening of 0.1 eV. The d-band center (ε_d) was calculated using Eq 1,

$$\varepsilon_{\mathrm{d}} = \frac{\int_{-\infty}^{E_{\mathrm{F}}} E\rho_{\mathrm{d}}(E)\mathrm{d}E}{\int_{-\infty}^{E_{\mathrm{F}}} \rho_{\mathrm{d}}(E)\mathrm{d}E} \qquad (1)$$

where ρ_{d} represents the density of states projected onto the target metal's d band and E_{f} is the Fermi energy [11].

4. Supplementary Figures and Tables

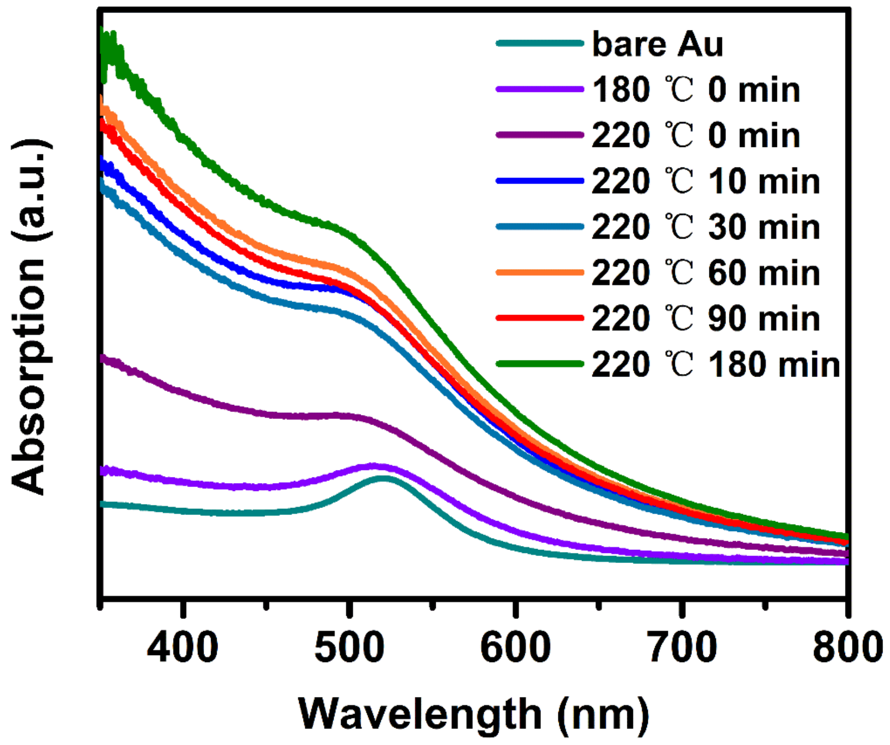

Figure S1. UV-visible absorption spectra of bare Au and Au@Au_xIr_{1-x} core-shell NPs obtained at different intervals during the reaction (180℃ 0 min; 220℃ 0 min, 10 min, 30min, 60min, 90min and 180min).

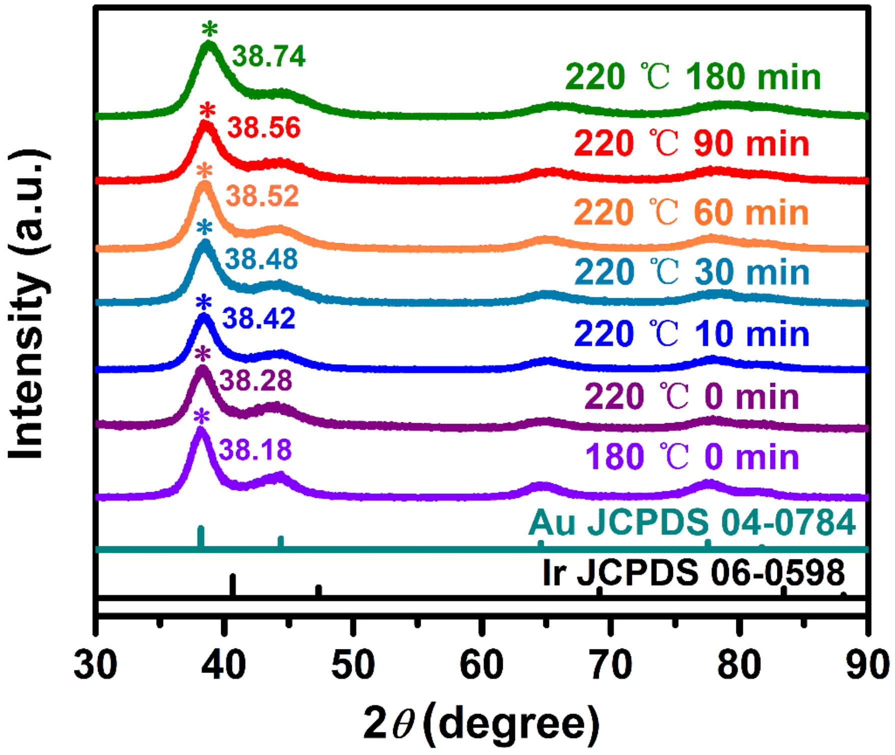

Figure S2. XRD patterns of $Au@Au_xIr_{1-x}$ core-shell NPs obtained at different intervals during the reaction (180°C 0 min; 220°C 0 min, 10 min, 30 min, 60 min, 90 min and 180 min). The standard XRD patterns of bulk gold (PDF# 04-0784) and iridium (PDF# 06-0598) are also shown in the panel for reference.

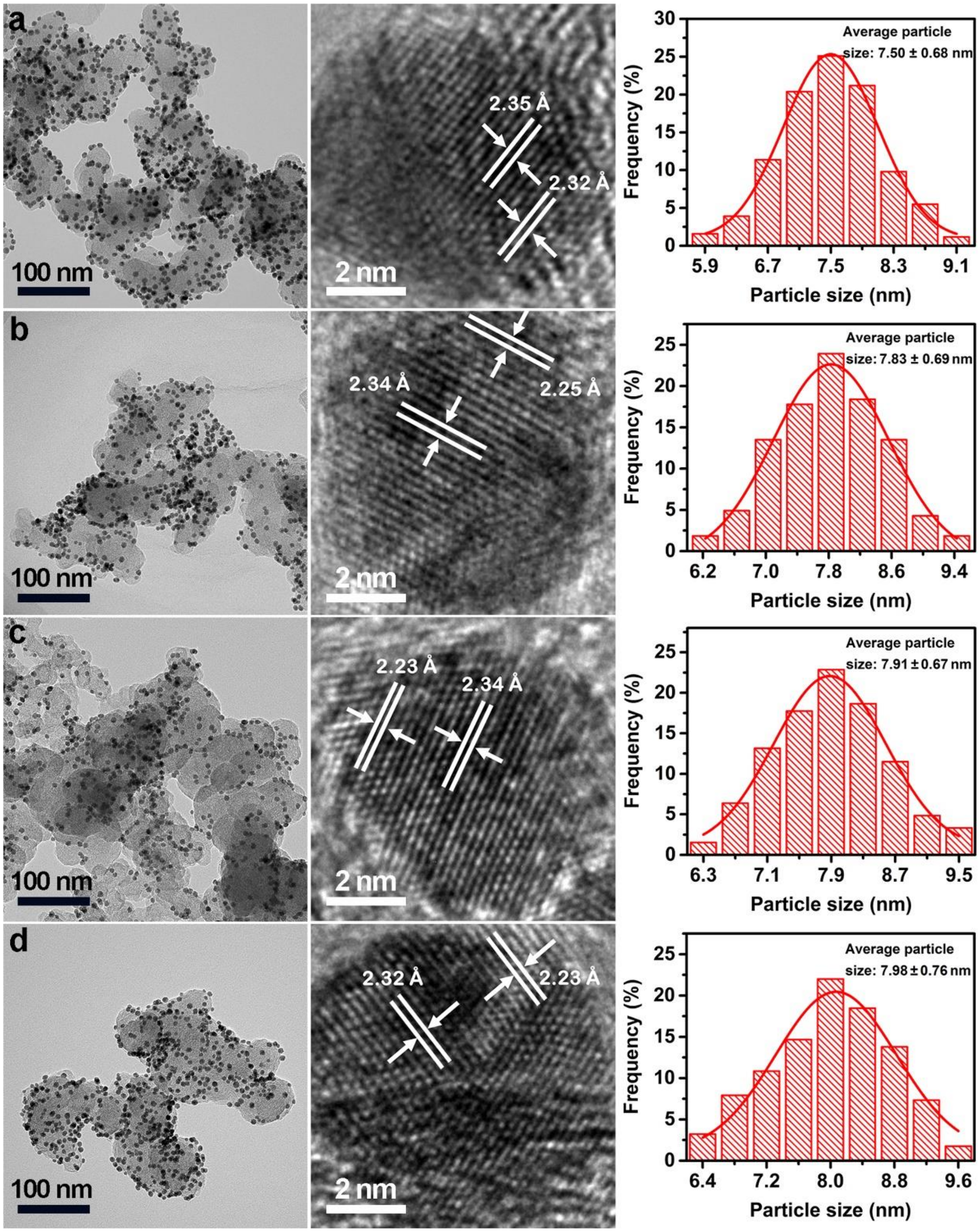

Figure S3. TEM images, HRTEM images and size distributions of (a) $Au@Au_{0.54}Ir_{0.46}$, (b) $Au@Au_{0.43}Ir_{0.57}$, (c) $Au@Au_{0.34}Ir_{0.66}$ and (d) $Au@Au_{0.26}Ir_{0.74}$ core-shell NPs.

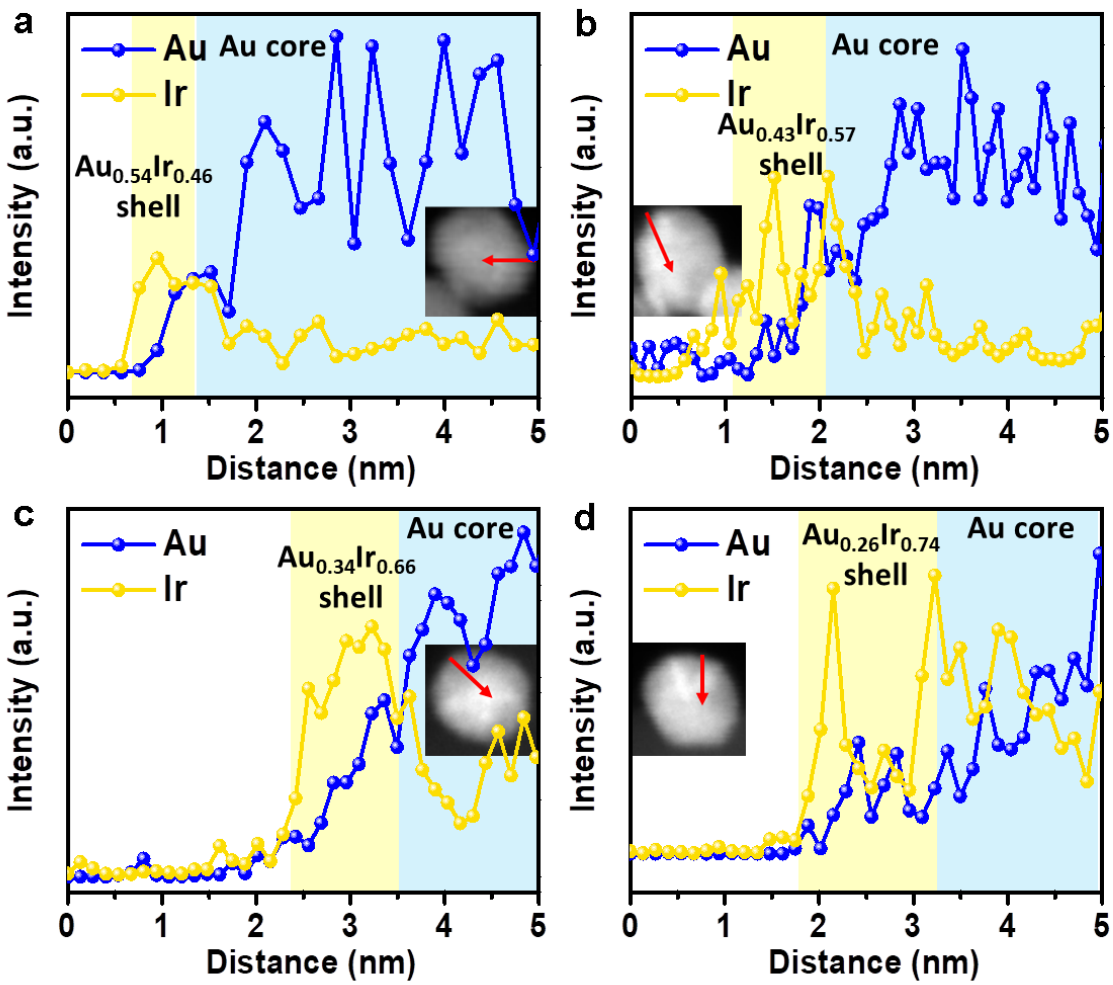

Figure S4. EDX line scan analyses of (a) $Au@Au_{0.54}Ir_{0.46}$, (b) $Au@Au_{0.43}Ir_{0.57}$, (c) $Au@Au_{0.34}Ir_{0.66}$ and (d) $Au@Au_{0.26}Ir_{0.74}$ along the red arrows marked in the insets.

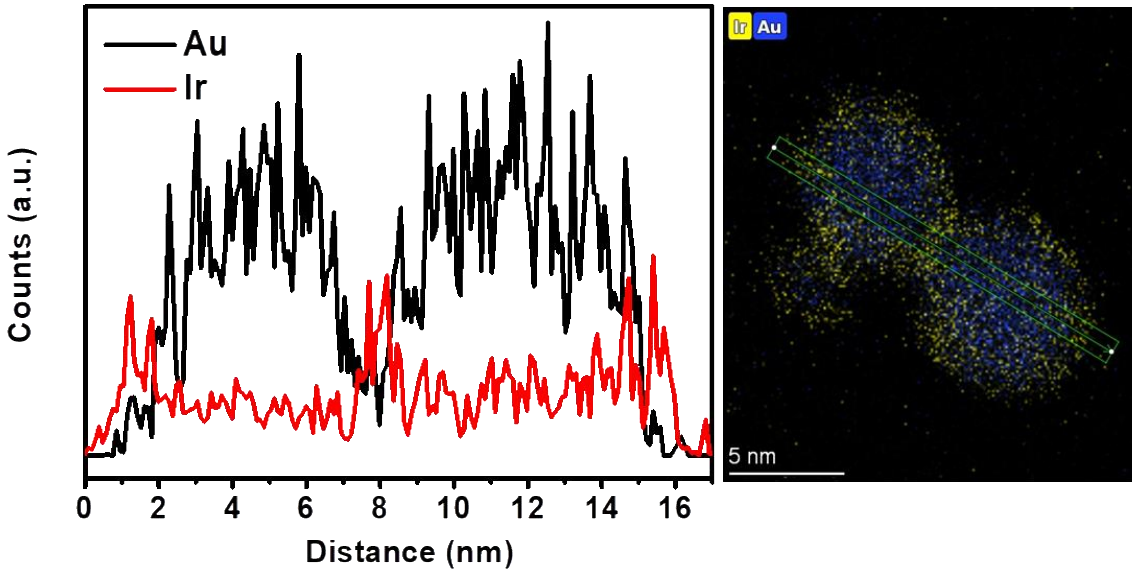

Figure S5. EDX line scan analysis of two random nanoparticles of $Au@Au_{0.43}Ir_{0.57}$.

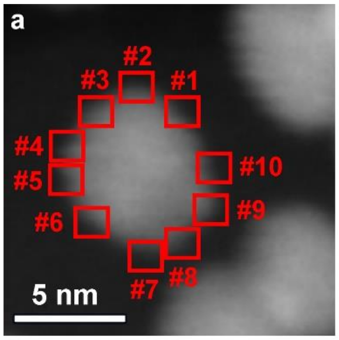

b

Position	Atomic Fraction (%)	
	Au	Ir
Area #1	53.12	46.88
Area #2	52.29	47.71
Area #3	56.88	43.12
Area #4	52.45	47.55
Area #5	57.55	42.45
Area #6	53.39	46.61
Area #7	54.08	45.92
Area #8	53.90	46.10
Area #9	50.47	49.53
Area #10	56.95	43.05
average	54.11	45.89

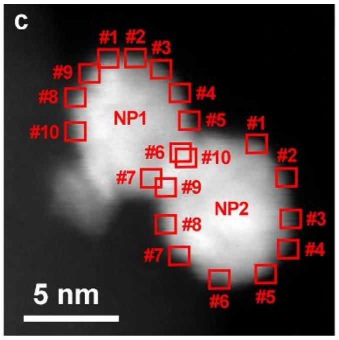

d

Particle	NP1		NP2	
Position	Atomic Fraction (%)		Atomic Fraction (%)	
	Au	Ir	Au	Ir
Area #1	41.34	58.66	41.50	58.5
Area #2	43.17	56.83	42.05	57.95
Area #3	42.94	57.06	42.99	57.01
Area #4	41.81	58.19	42.00	58.00
Area #5	43.85	56.15	43.02	56.98
Area #6	39.86	60.14	42.28	57.72
Area #7	43.16	56.84	42.45	57.55
Area #8	45.67	54.33	42.98	57.02
Area #9	44.76	55.24	44.72	55.28
Area #10	48.19	51.81	39.50	60.50
average	43.47	56.53	42.35	57.65

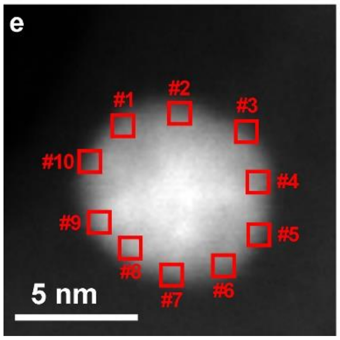

f

Position	Atomic Fraction (%)	
	Au	Ir
Area #1	33.26	66.74
Area #2	34.15	65.85
Area #3	34.60	65.40
Area #4	33.03	66.97
Area #5	36.09	63.91
Area #6	33.29	66.71
Area #7	33.25	66.75
Area #8	34.65	65.35
Area #9	33.79	66.21
Area #10	32.56	67.44
average	33.87	66.13

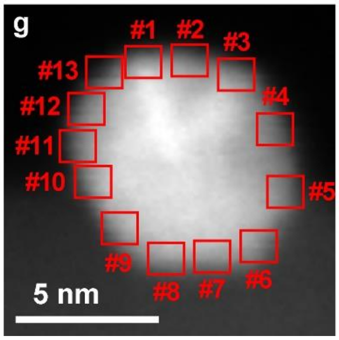

h

Position	Atomic Fraction (%)	
	Au	Ir
Area #1	32.66	67.34
Area #2	19.01	80.99
Area #3	17.64	82.36
Area #4	35.98	64.02
Area #5	36.32	63.68
Area #6	33.05	66.95
Area #7	29.66	70.34
Area #8	21.83	78.17
Area #9	29.87	70.13
Area #10	18.14	81.86
Area #11	28.28	71.72
Area #12	17.60	82.40
Area #13	21.32	78.68
average	26.26	73.74

Figure S6. The HAADF-STEM images of (a) $Au@Au_{0.54}Ir_{0.46}$, (c) $Au@Au_{0.43}Ir_{0.57}$, (e) $Au@Au_{0.34}Ir_{0.66}$ and (g) $Au@Au_{0.26}Ir_{0.74}$; The atomic ratios on different positions, which were estimated by EDS analysis: (b) $Au@Au_{0.54}Ir_{0.46}$, (d) $Au@Au_{0.43}Ir_{0.57}$, (f) $Au@Au_{0.34}Ir_{0.66}$ and (h) $Au@Au_{0.26}Ir_{0.74}$.

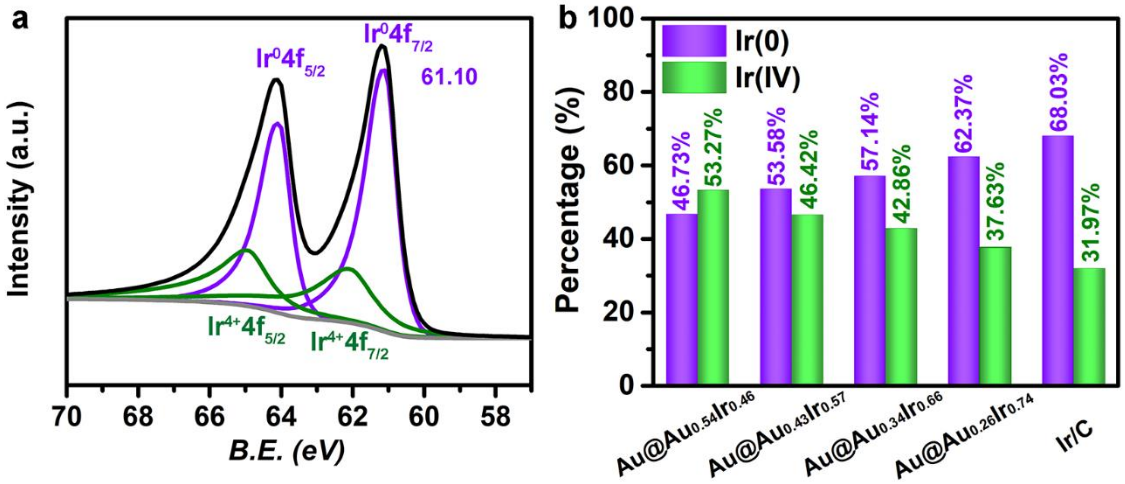

Figure S7. (a) XPS spectrum of commercial Ir/C, (b) IrO_x percentage (estimated by XPS) of $Au@Au_xIr_{1-x}$ and commercial Ir/C.

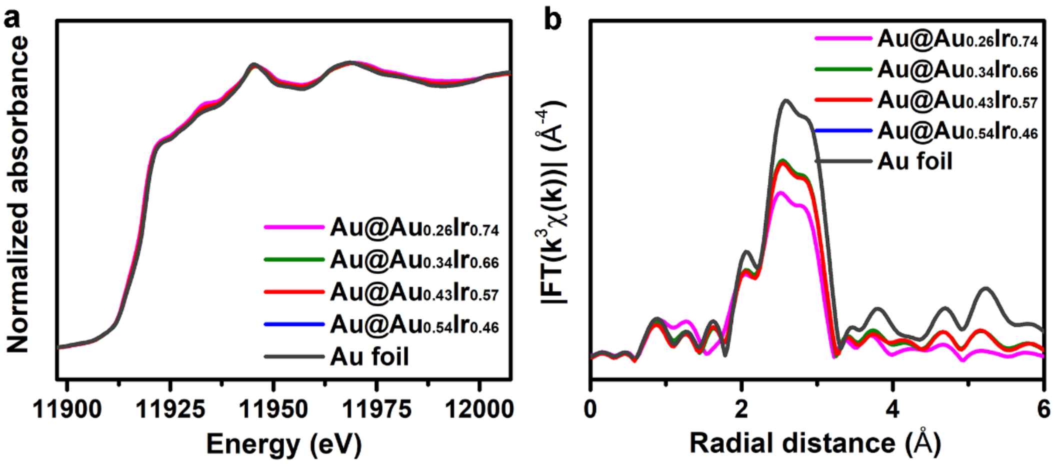

Figure S8. (a) Au L_3-edge XANES spectra, (b) k^3-weighted Fourier-transform L_3-edge EXAFS spectra of $Au@Au_xIr_{1-x}$ and Au foil.

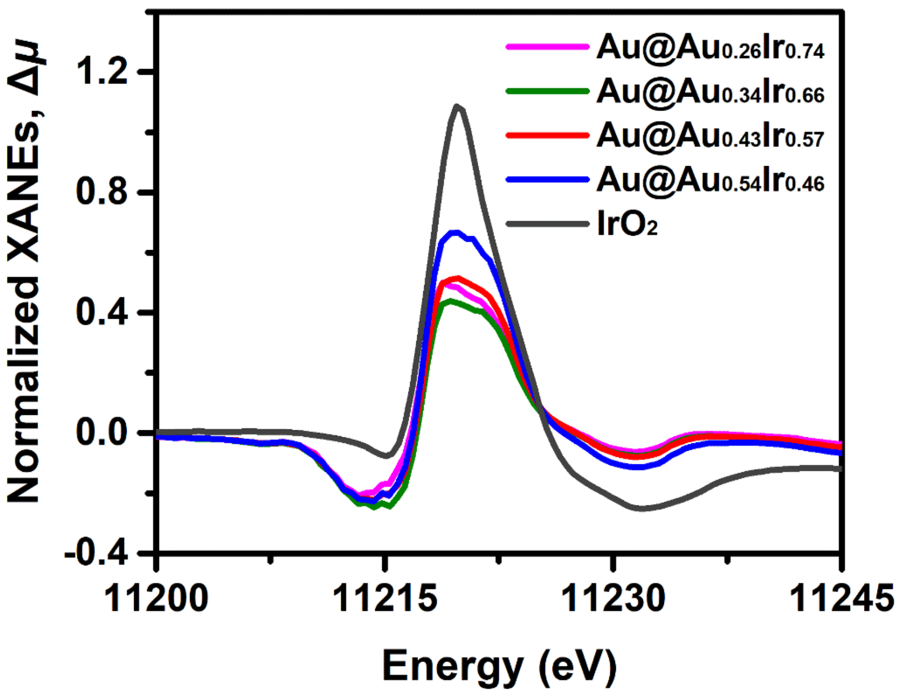

Figure S9. Normalized difference spectra for Ir L_3-edge XANES using Ir powder as reference. The valence states are fitted through integrating the area of the white-line peak from 11215.1 to 11231.5 eV.

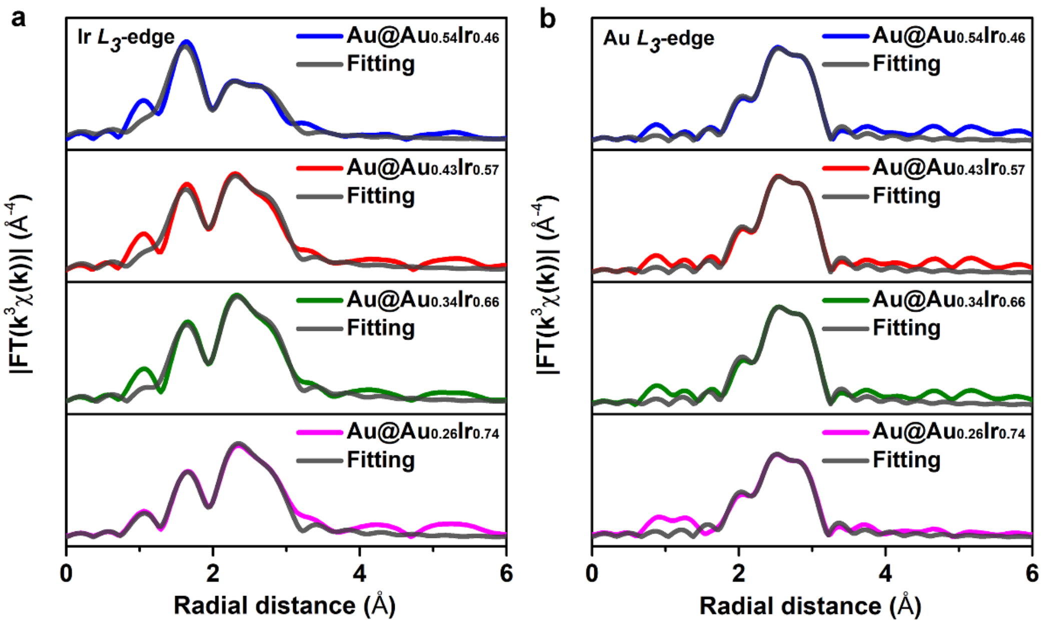

Figure S10. The fitting Ir L_3-edge EXAFS spectra (a) and Au L_3-edge EXAFS spectra (b) of Au@Au_xIr_{1-x}.

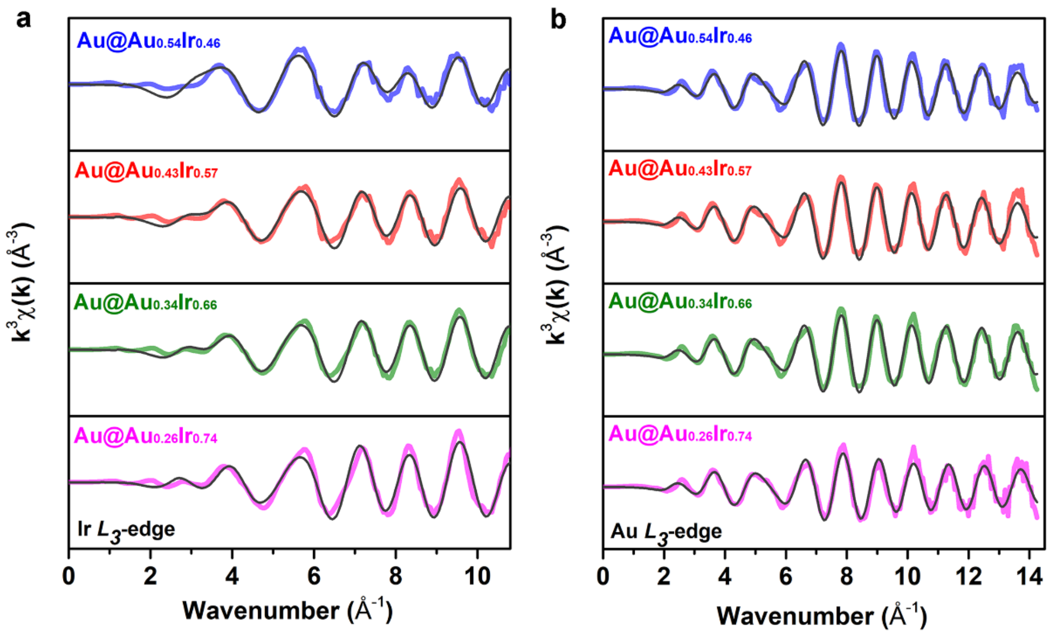

Figure S11. Corresponding k^3-weighted EXAFS spectra of Ir L_3-edge EXAFS spectra (a) and Au L_3-edge EXAFS spectra (b) of the $Au@Au_xIr_{1-x}$. The black lines show the fitting data of each EXAFS spectrum.

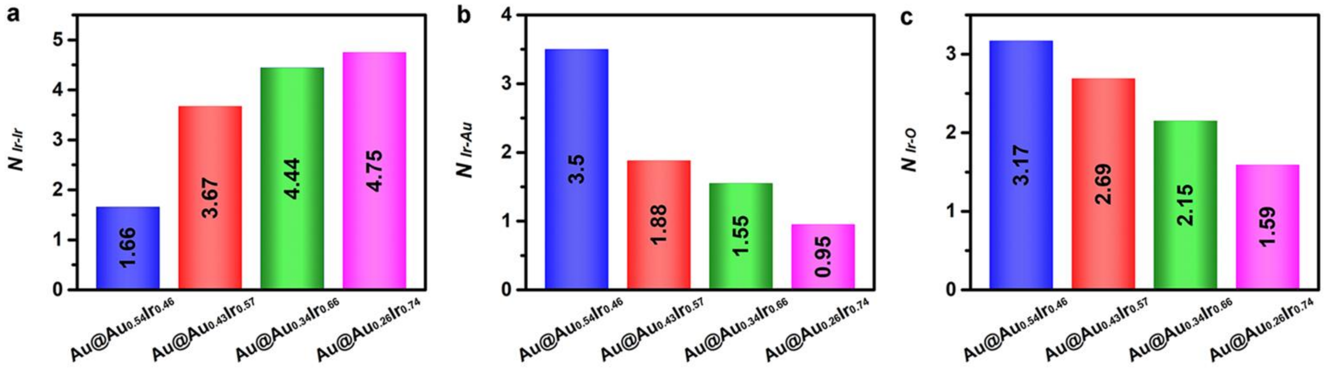

Figure S12. The coordination number of (a) Ir─Ir (N_{Ir-Ir}), (b) Ir─Au (N_{Ir-Au}) and (c) Ir─O (N_{Ir-O}) for Au@Au$_x$Ir$_{1-x}$.

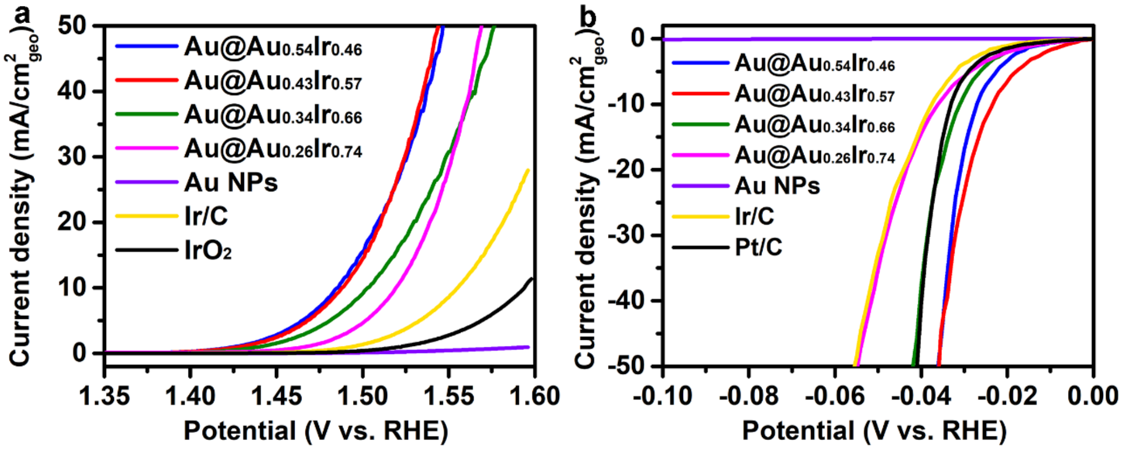

Figure S13. (a) OER polarization curves of Au@Au_xIr_{1-x}, commercial Ir/C and IrO_2 catalysts with Ir loading of 10.2 $\mu g_{Ir}/cm^2$ on RDE. (b) HER polarization curves of Au@Au_xIr_{1-x}, commercial Ir/C and Pt/C catalysts with metal loading of 10.2 $\mu g_{Ir/Pt}/cm^2$ on RDE.

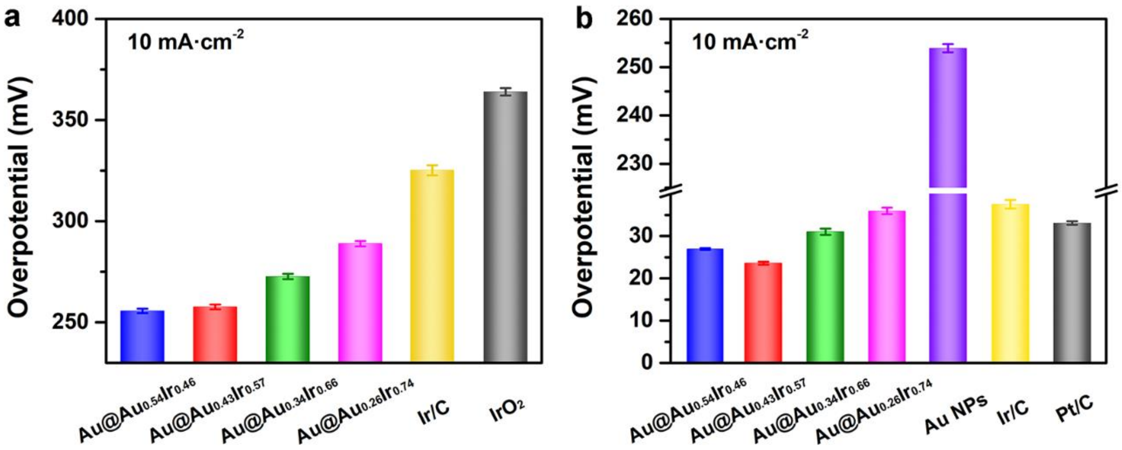

Figure S14. The overpotentials reach to 10 mA/cm^2 for OER (a) and HER (b). The test has been performed at least five times and gives the shown error bars.

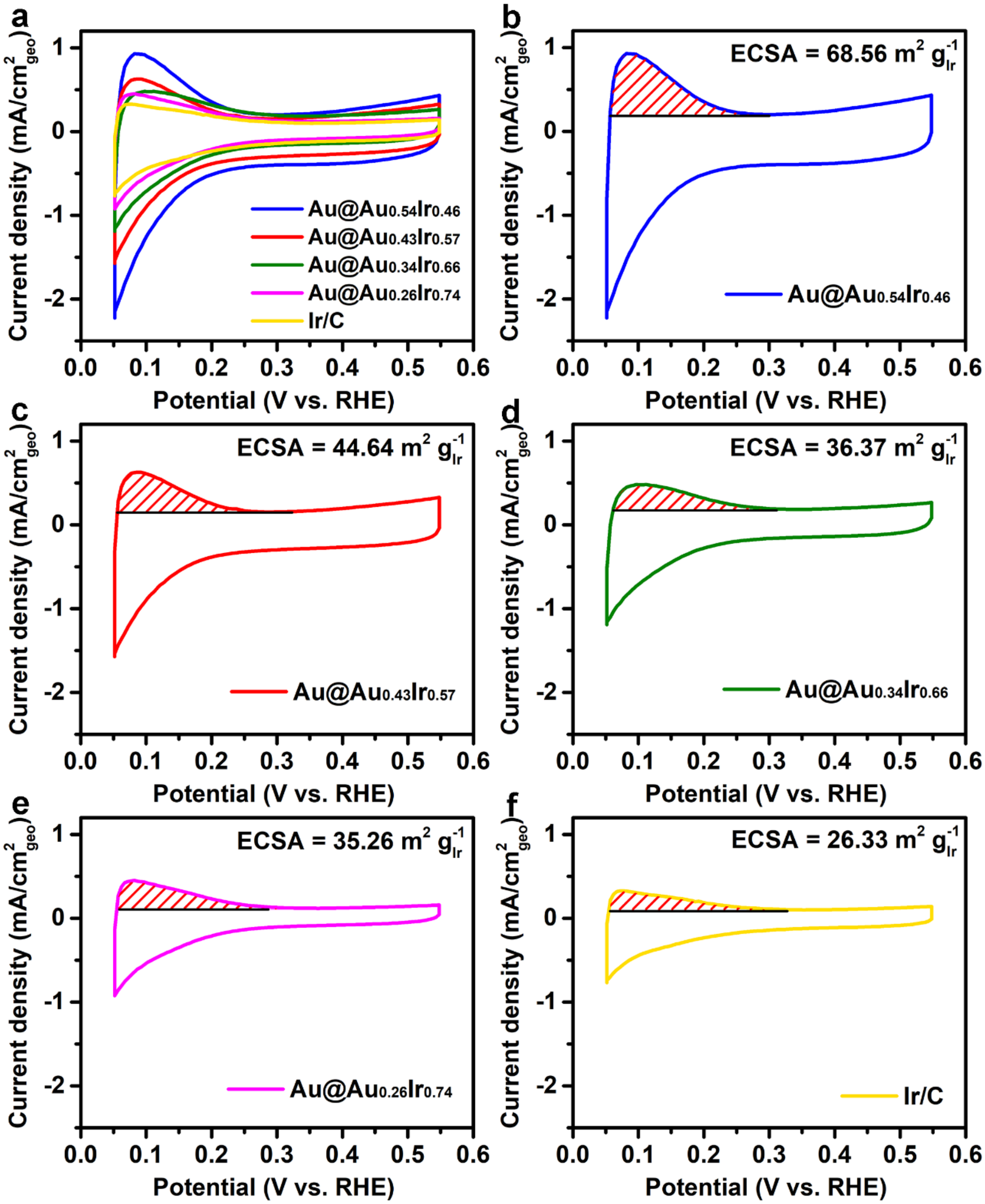

Figure S15. (a) CVs of Au@Au$_x$Ir$_{1-x}$ in 0.5 M H_2SO_4 performed between 0.05 and 0.55 V vs. RHE. Their corresponding specific ECSAs were calculated by H_{upd} area (b) Au@Au$_{0.54}$Ir$_{0.46}$, (c) Au@Au$_{0.43}$Ir$_{0.57}$, (d) Au@Au$_{0.34}$Ir$_{0.66}$, (e) Au@Au$_{0.26}$Ir$_{0.74}$ and (f) Ir/C [12-14].

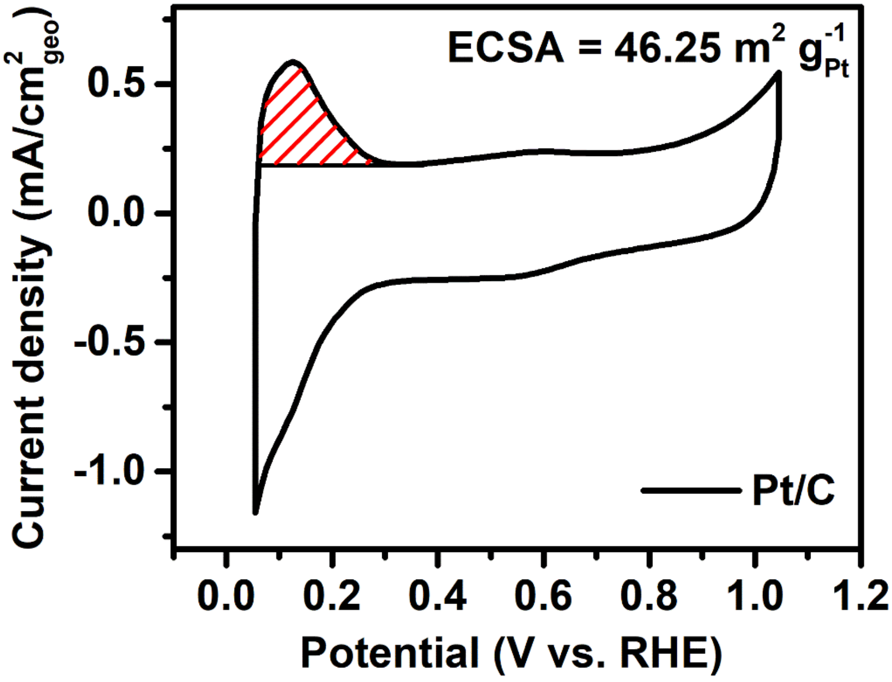

Figure S16. CV of Pt/C in 0.5 M H_2SO_4 performed between 0.05 and 1.05 V vs RHE and corresponding specific ECSA calculated by H_{upd} area.

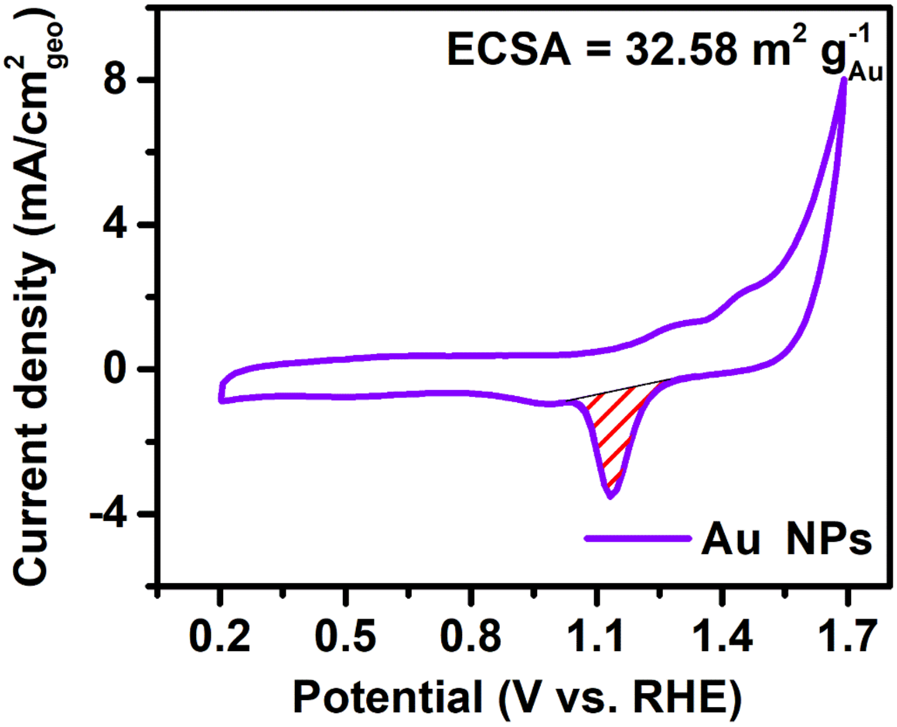

Figure S17. CV of Au measured between 0.2 and 1.7 V in 0.5 M H_2SO_4 and corresponding specific ECSA calculated by the reduction peak charge of Au oxides.

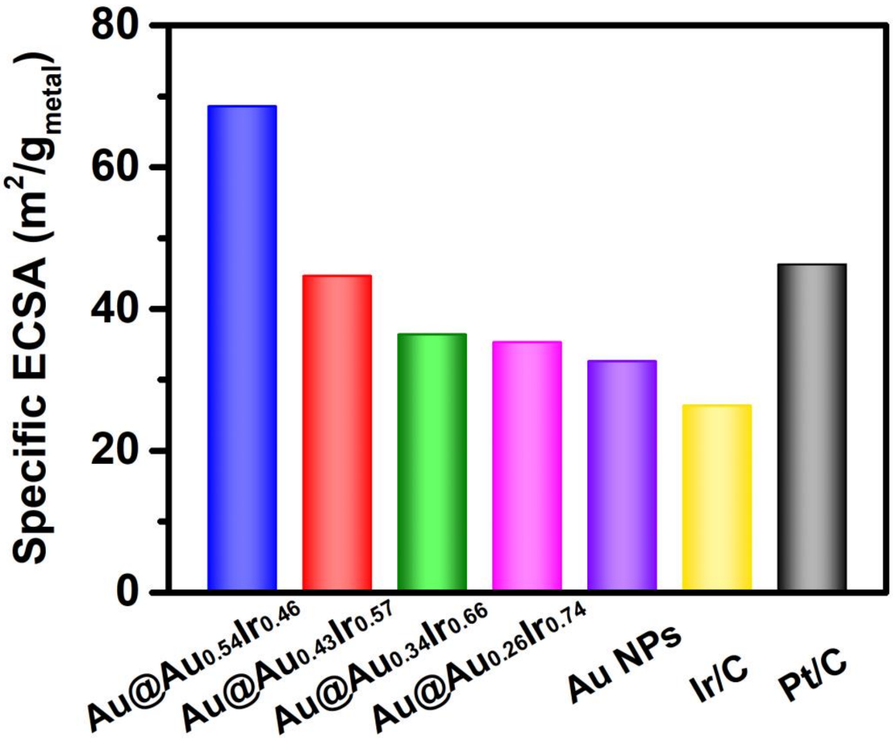

Figure S18. Specific ECSAs of Au@Au_xIr_{1-x}, commercial Ir/C and Pt/C catalysts at loading of 10.2 $\mu g_{Ir/Pt}/cm^2$ on RDE.

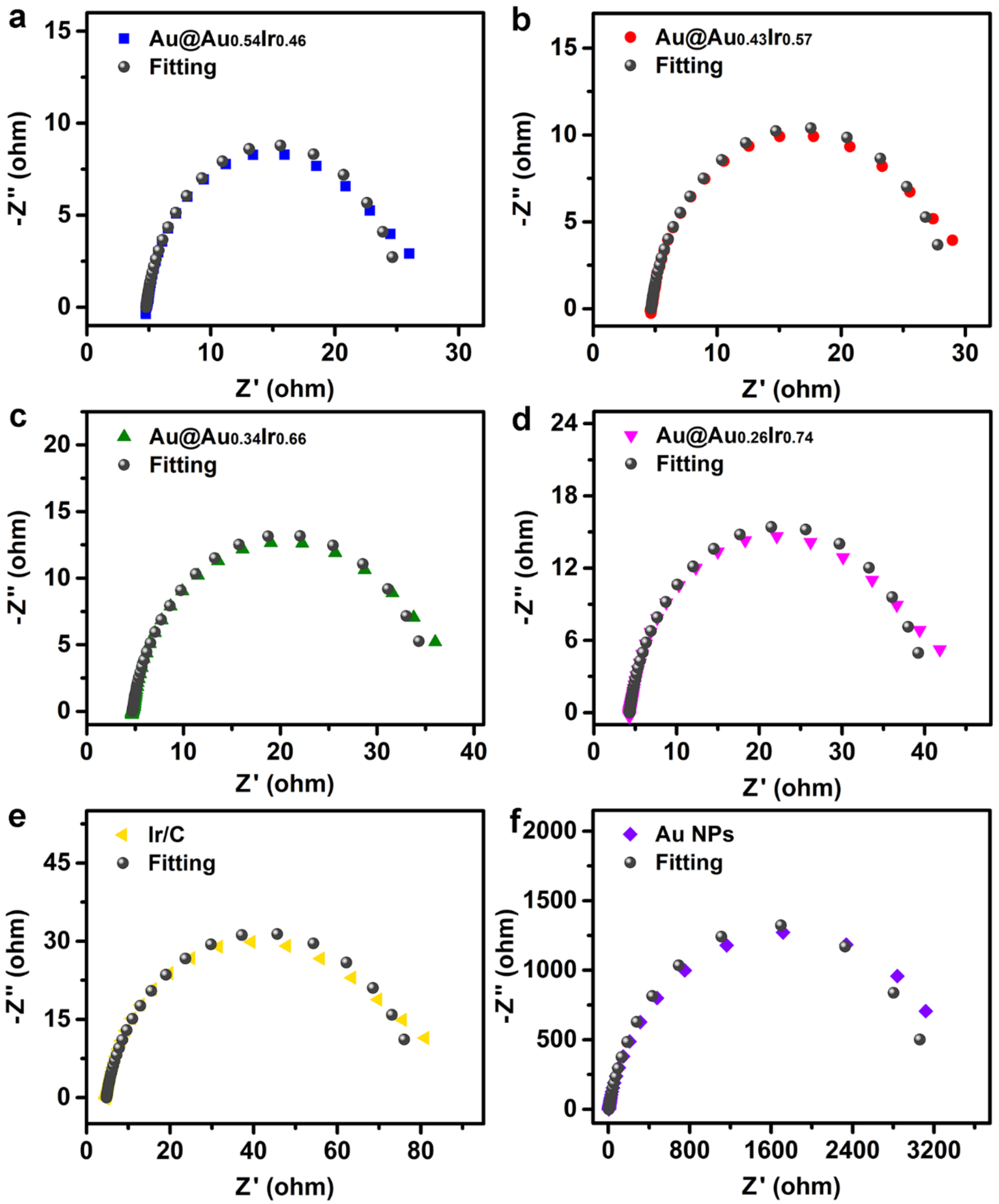

Figure S19. EIS measurements and their fitting data of (a) $Au@Au_{0.54}Ir_{0.46}$, (b) $Au@Au_{0.43}Ir_{0.57}$, (c) $Au@Au_{0.34}Ir_{0.66}$, (d) $Au@Au_{0.26}Ir_{0.74}$ (e) Ir/C and (f) Au at 1.52 V with a frequency range of 100 kHz–10 mHz.

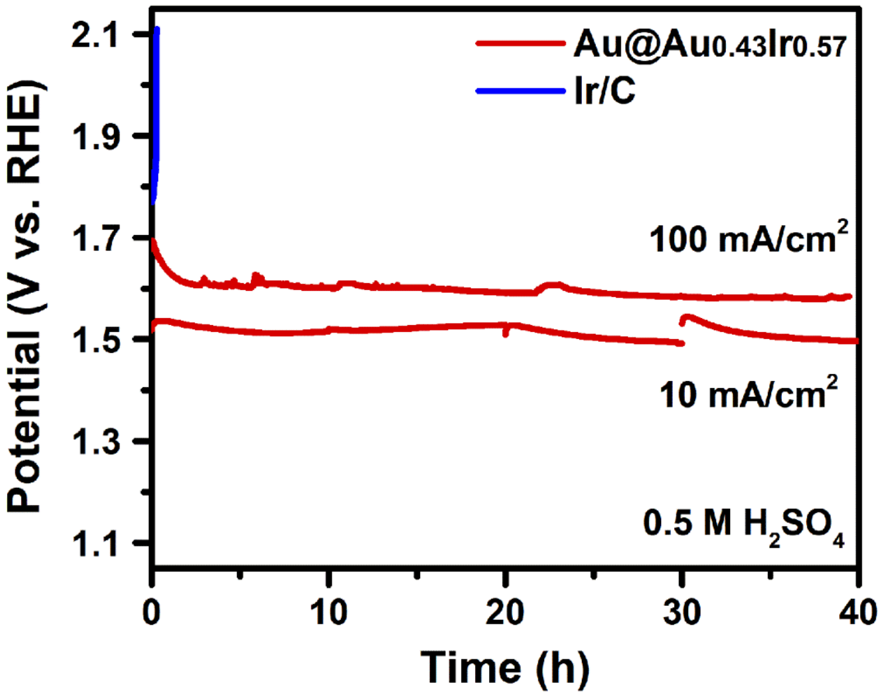

Figure S20. Chronopotentiometric test for OER at 10 mA/cm^2 of Au@Au$_{0.43}$Ir$_{0.57}$, 100 mA/cm^2 of Au@Au$_{0.43}$Ir$_{0.57}$ and Ir/C.

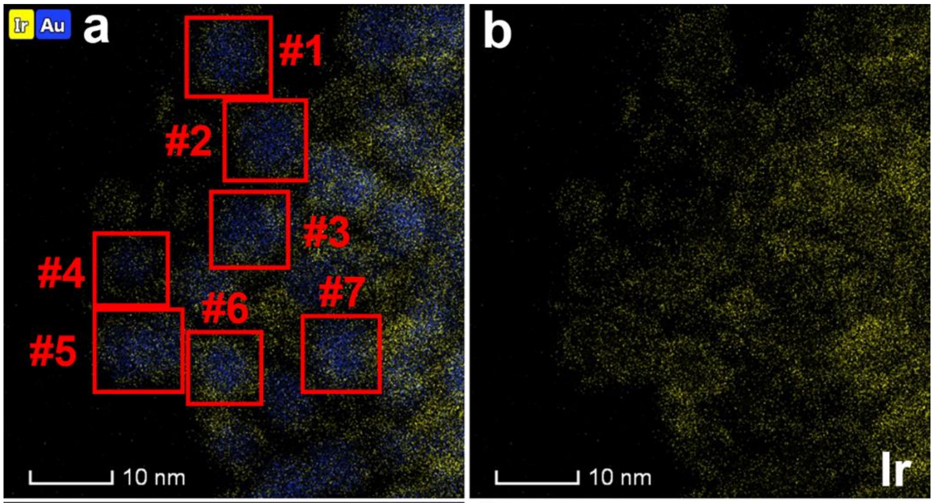

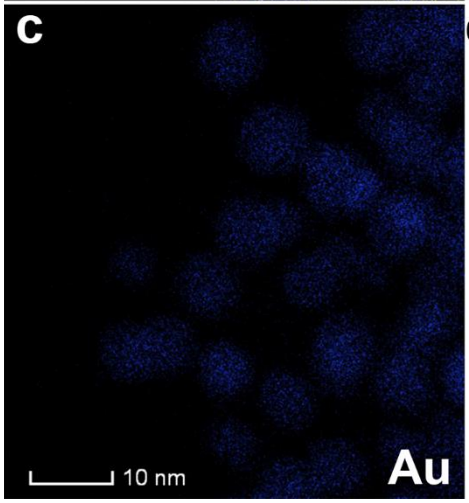

d

Particle	Total Atomic Fraction (%)		Shell Atomic Fraction (%)	
	Au	Ir	Au	Ir
Particle #1	71.69	28.31	42.21	57.79
Particle #2	71.86	28.14	40.65	59.35
Particle #3	72.21	27.79	44.38	55.62
Particle #4	71.58	28.42	43.13	56.87
Particle #5	72.45	27.55	43.85	56.15
Particle #6	72.17	27.83	43.58	56.42
Particle #7	72.29	27.71	44.85	55.15
Average	72.04	27.96	43.24	56.76

Figure S21. STEM and the shell composition of $Au@Au_{0.43}Ir_{0.57}$ after the OER test.

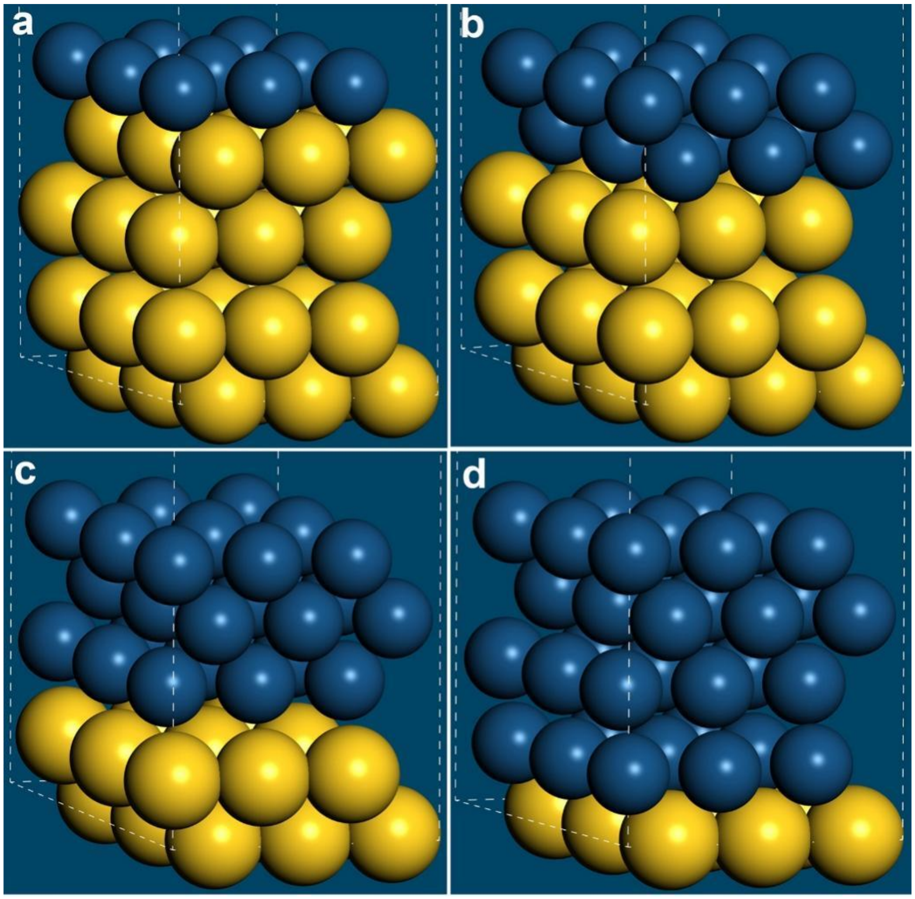

Figure S22. DFT calculation models of (a) Ir_9Au_{36}, (b) $Ir_{18}Au_{27}$, (c) $Ir_{27}Au_{18}$ and (d) $Ir_{36}Au_9$.

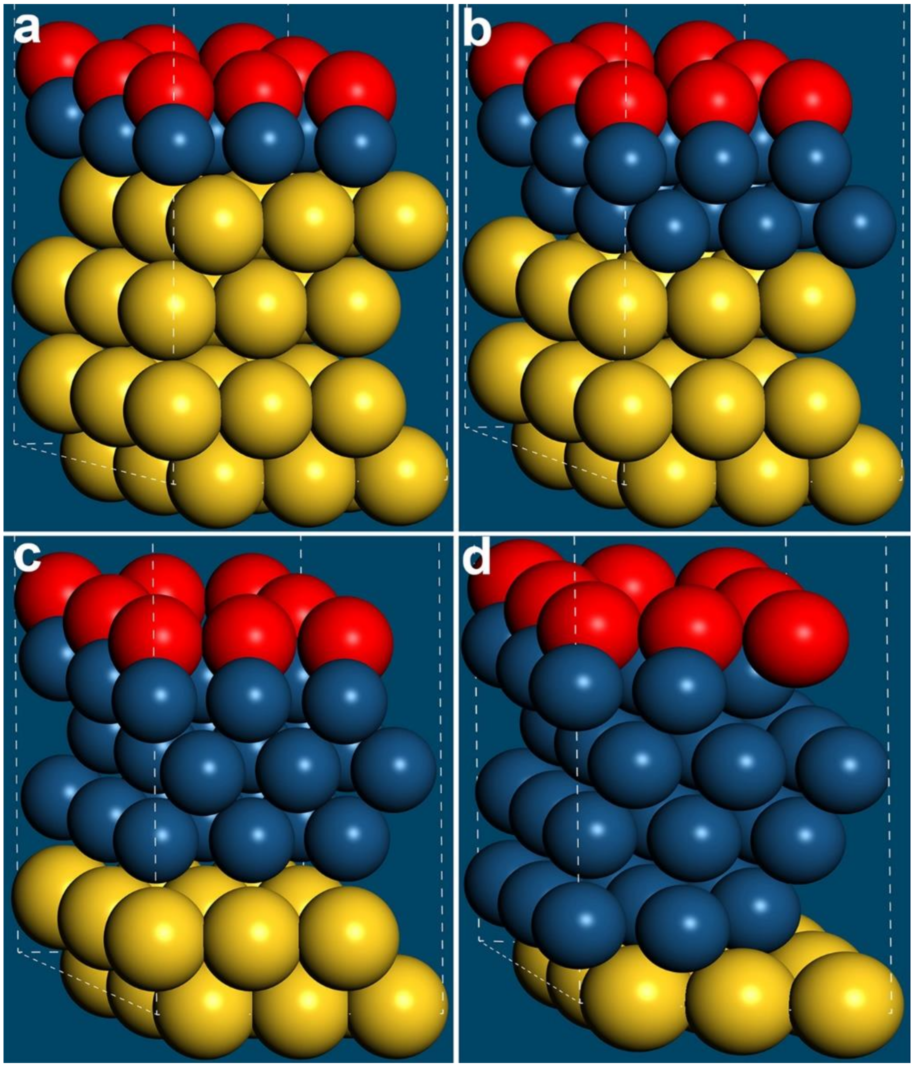

Figure S23. DFT calculation models of (a) $Ir_9Au_{36}O_8$, (b) $Ir_{18}Au_{27}O_8$, (c) $Ir_{27}Au_{18}O_8$ and (d) $Ir_{36}Au_9O_8$.

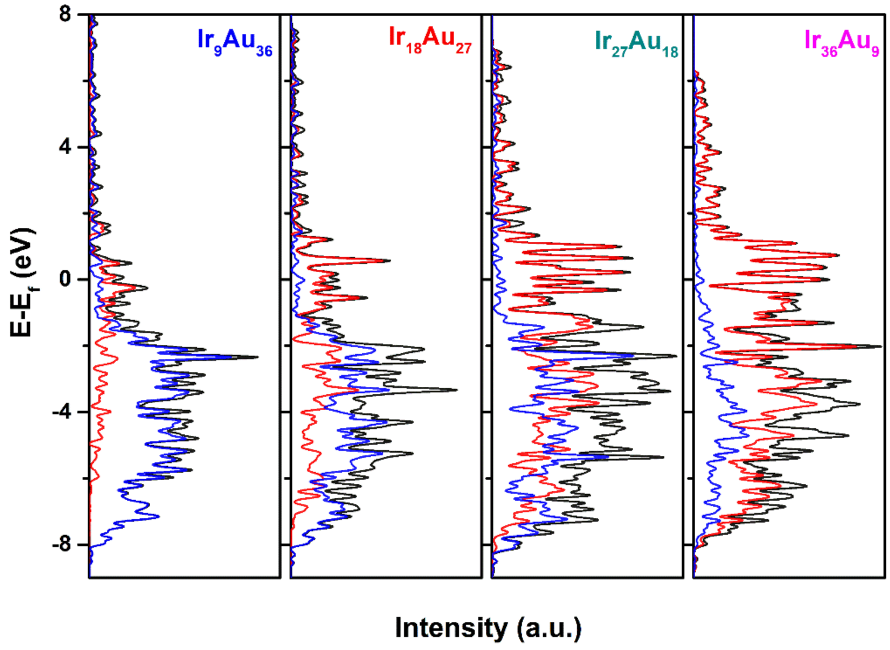

Figure S24. The electronic structures of the catalyst surfaces studied by DFT calculations. DOS of Au_xIr_y (111) surfaces, blue and red lines indicate the Au and Ir LDOS, respectively.

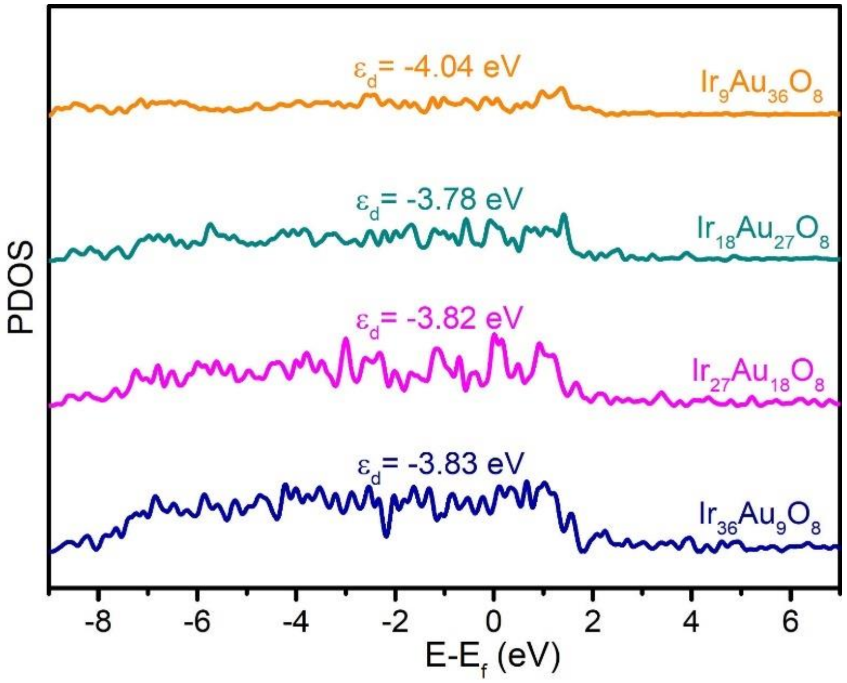

Figure S25. DFT calculated PDOS of *d*-bands and *d*-band centers of Ir atoms for $Au_xIr_yO_z$ systems.

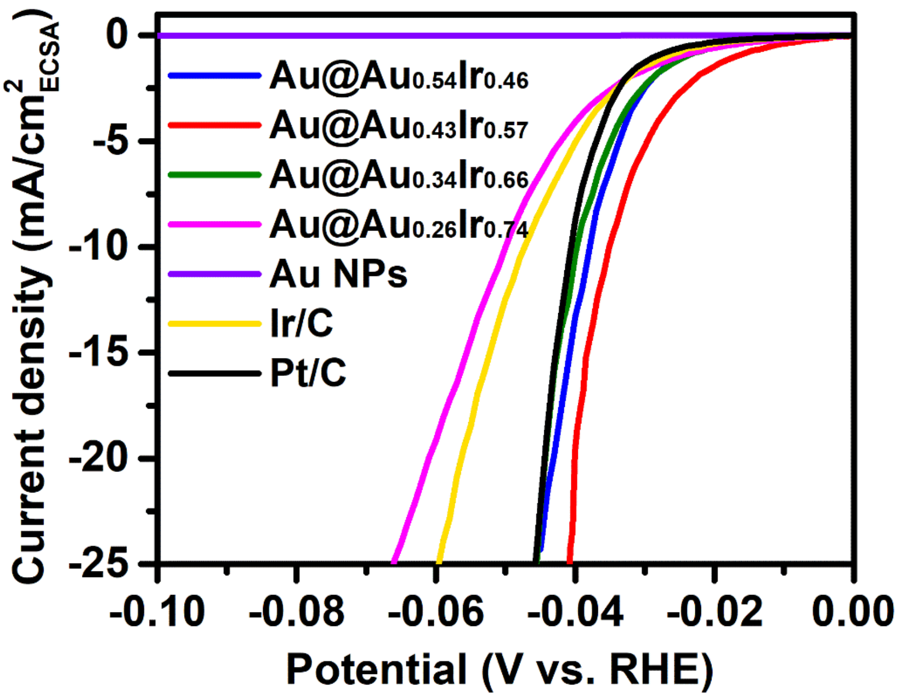

Figure S26. HER polarization curves normalized by ECSA of Au@Au_xIr_{1-x}, commercial Ir/C and Pt/C in 0.5 M H_2SO_4 solution.

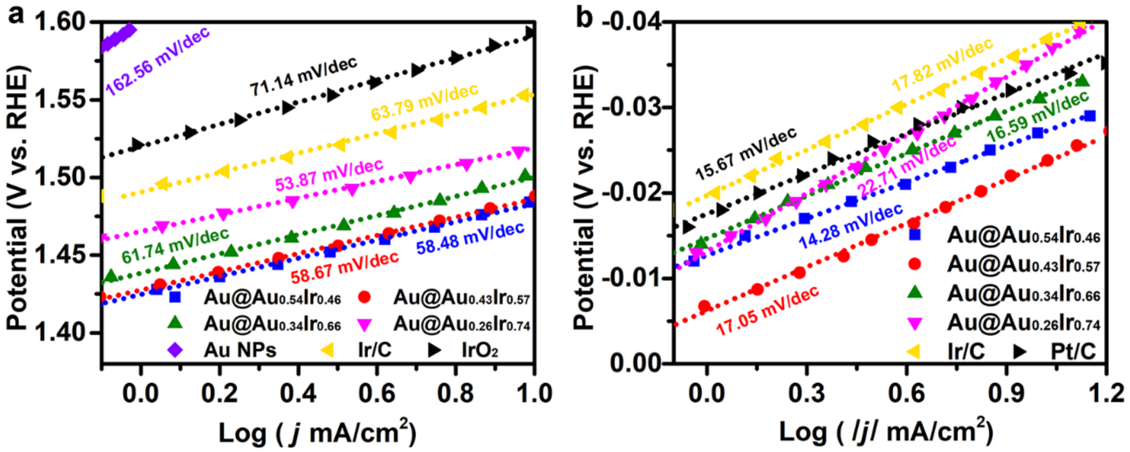

Figure S27. Tafel plots derived from (a) OER and (b) HER polarization curves.

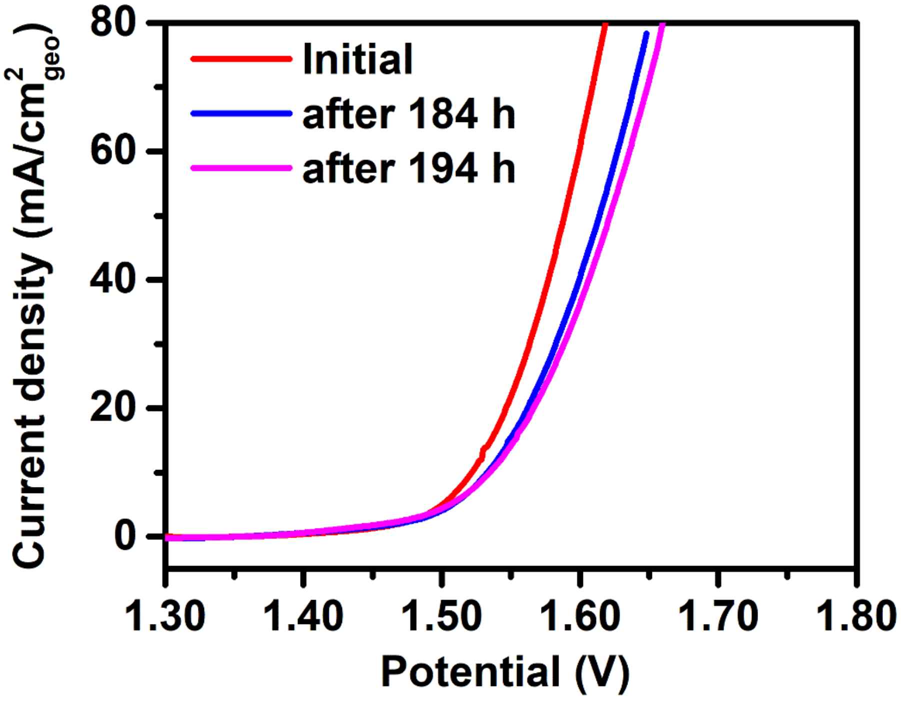

Figure S28. LSV curves before and after durability test of Au@$Au_{0.43}Ir_{0.57}$.

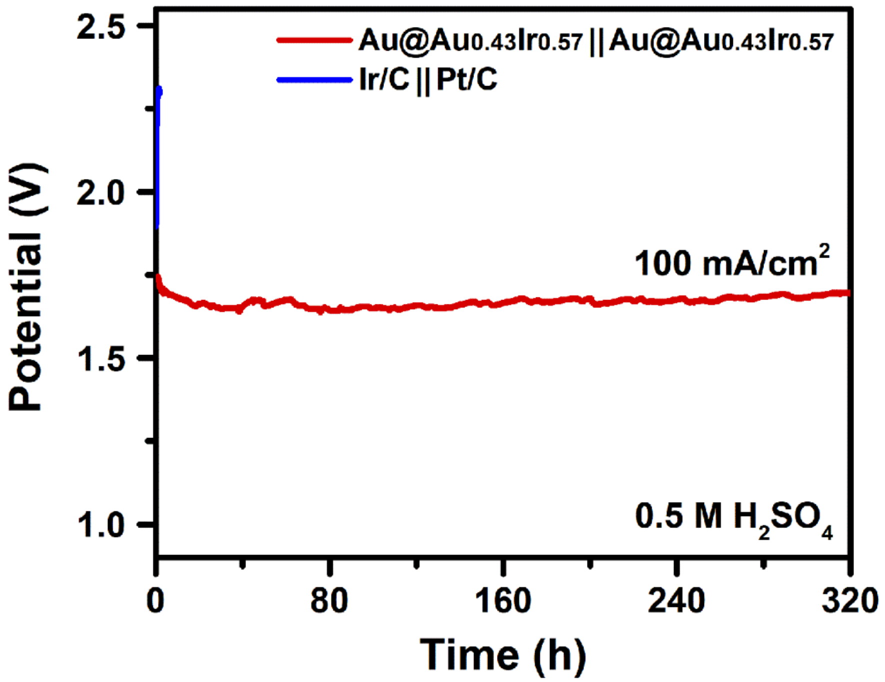

Figure S29. Chronopotentiometric test at 100 mA/cm^2 for overall water splitting of Au@Au$_{0.43}$Ir$_{0.57}$ || Au@Au$_{0.43}$Ir$_{0.57}$ and Ir/C || Pt/C.

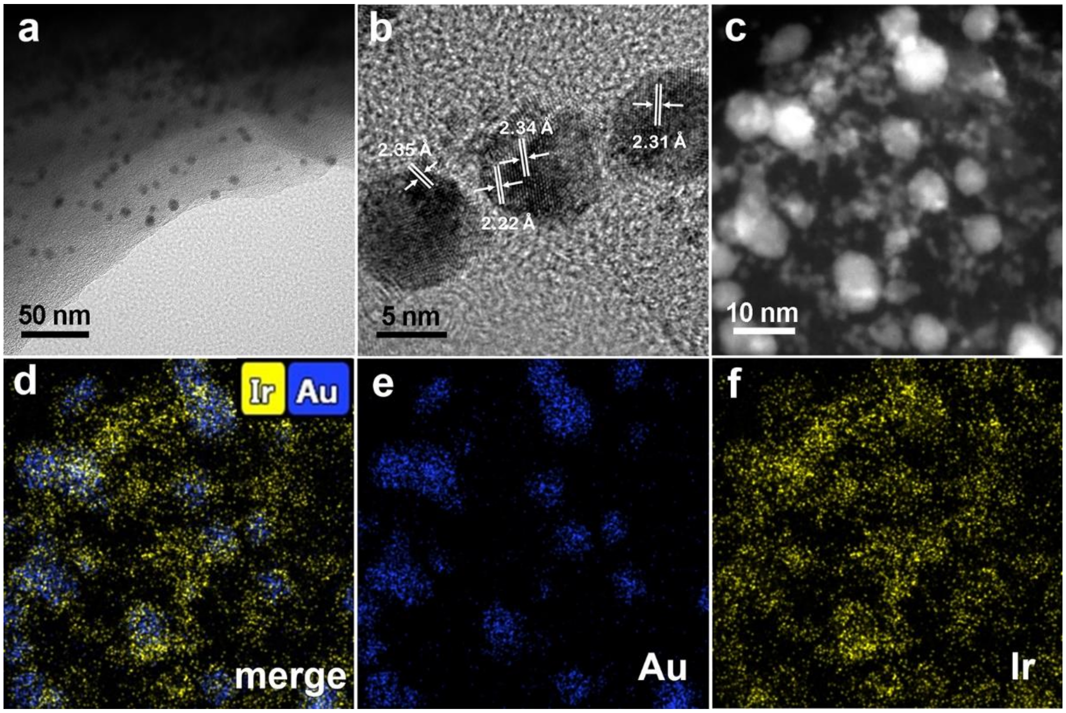

Figure S30. TEM, HRTEM, HAADF-STEM images of $Au@Au_{0.43}Ir_{0.57}$ after durability.

Supplementary Tables

Table S1 Atomic ratios of as-synthesized Au@Au_xIr_{1-x} core-shell nanoparticles were obtained at different intervals during the reaction by ICP-OES and EDS.

Sample (%)	$HAuCl_4•3H_2O$ (mmol)	$IrCl_3•xH_2O$ (mmol)	Experiment condition	ICP-OES		EDS	
				Au	Ir	Au	Ir
Au	0.1334	0.0667	180℃ 0 min	99.28	0.72	99.23	0.77
Au@$Au_{0.54}Ir_{0.46}$	0.1334	0.0667	220℃ 10 min	75.25	24.75	76.30	23.70
Au@$Au_{0.48}Ir_{0.52}$	0.1334	0.0667	220℃ 30 min	74.12	25.88	74.16	25.84
Au@$Au_{0.45}Ir_{0.55}$	0.1334	0.0667	220℃ 60 min	72.37	27.63	72.34	27.66
Au@$Au_{0.43}Ir_{0.57}$	0.1334	0.0667	220℃ 90 min	70.99	29.01	71.65	28.35
Au@$Au_{0.34}Ir_{0.66}$	0.1334	0.0667	220℃ 180 min	67.90	32.10	67.97	32.03
Au@$Au_{0.26}Ir_{0.74}$	0.1334	0.0800	220℃ 180 min	63.40	36.60	64.69	35.31

Table S2 Average charge of surface Ir atoms in Au_xIr_y counted by DFT calculations.

Sample	Charge of surface Ir atoms (eV)
Ir_9Au_{36}	-0.28
$Ir_{18}Au_{27}$	-0.15
$Ir_{27}Au_{18}$	-0.56
$Ir_{36}Au_9$	-0.50

Table S3 Fitting Results of EXAFS Data at Au L_3-Edge and Ir L_3-Edge.

sample	bond	CN	bond length (Å)	σ^2 (10^{-3} Å^2)	ΔE_0 (eV)	r-factor
Au foil	Au-Au	12.00	2.86	8.05	1.20	0.0081
$Au@Au_{0.54}Ir_{0.46}$	Au-Au	7.42	2.85	9.40	1.69	0.0021
	Au-Ir	3.03	2.79	6.21	1.69	0.0021
	Ir-Au	3.50	2.79	7.93	6.47	0.0126
	Ir-Ir	1.66	2.70	6.23	6.47	0.0126
	Ir-O	3.17	2.00	7.42	6.47	0.0126
$Au@Au_{0.43}Ir_{0.57}$	Au-Au	7.40	2.85	9.33	1.66	0.0022
	Au-Ir	3.20	2.79	6.10	1.66	0.0022
	Ir-Au	1.88	2.79	23.80	4.94	0.0068
	Ir-Ir	3.67	2.69	7.58	4.94	0.0068
	Ir-O	2.69	1.98	7.62	4.94	0.0068
$Au@Au_{0.34}Ir_{0.66}$	Au-Au	7.55	2.84	10.17	1.57	0.0018
	Au-Ir	3.29	2.80	20.13	1.57	0.0018
	Ir-Au	1.55	2.80	20.13	5.27	0.0052
	Ir-Ir	4.44	2.70	7.26	5.27	0.0052
	Ir-O	2.15	1.98	5.00	5.27	0.0052
$Au@Au_{0.26}Ir_{0.74}$	Au-Au	6.08	2.82	12.49	0.60	0.0011
	Au-Ir	5.03	2.81	6.31	0.60	0.0011

	Ir-Au	0.95	2.81	9.80	3.88	0.0060
	Ir-Ir	4.75	2.69	6.95	3.88	0.0060
	Ir-O	1.59	1.98	2.95	3.88	0.0060
Ir (fcc)	Ir-Ir	12.00*	2.74	-	-	-
IrO_2	Ir-O	5.60	1.98	2.14	9.20	0.0045

N, coordination number; *R*, interatomic distance; σ^2, Debye-Waller factor; Error bounds (accuracies) were estimated as *N*, ±10%; *R*, ±1%; σ^2, ±10%. * is fixed coordination number according to the standard crystal structure. For comparison, the expected interatomic distances of reference systems based on their standard crystal structures are presented here: Ir (fcc): Ir-Ir, 2.74 Å; IrO_2: Ir-O, 2.00 Å; Au foil: Au-Au, 2.86 Å.

Table S4 Mass loading of $Au@Au_xIr_{1-x}$ catalysts for OER and HER.

Sample	Ir (μg/cm^2)	Au (μg/cm^2)	Total (μg/cm^2)
$Au@Au_{0.54}Ir_{0.46}$	10.20	31.01	41.21
$Au@Au_{0.43}Ir_{0.57}$	10.20	24.96	35.16
$Au@Au_{0.34}Ir_{0.66}$	10.20	21.58	31.78
$Au@Au_{0.26}Ir_{0.74}$	10.20	17.67	27.87

Table S5 OER performances of $Au@Au_xIr_{1-x}$ compared with recently reported Ir-based catalysts in acidic electrolytes.

Catalysts	Electrolyte	Mass loading ($\mu g_{Ir}/cm^2$)	η (mV) to 10 mA/cm^2	Mass activity at η = 300 mV (A/mg)	*iR*-corrected condition	Ref.
$Au@Au_{0.54}Ir_{0.46}$	0.5 M H_2SO_4	10.2	255	3.259	90%	This work
$Au@Au_{0.43}Ir_{0.57}$	0.5 M H_2SO_4	10.2	257	3.402	90%	This work
$Au@Au_{0.34}Ir_{0.66}$	0.5 M H_2SO_4	10.2	273	1.978	90%	This work
$Au@Au_{0.26}Ir_{0.74}$	0.5 M H_2SO_4	10.2	289	1.431	90%	This work
$IrO_x/SrIrO_3$	0.5 M H_2SO_4	N.A.	270	N.A.	100%	[15]
$Pd@Ir_{1L}/C$	0.1 M $HClO_4$	10.2	300	0.950	95%	[16]
$Pd@Ir_{2L}/C$	0.1 M $HClO_4$	10.2	286	1.590	95%	[16]
$Pd@Ir_{3L}/C$	0.1 M $HClO_4$	10.2	263	3.330	95%	[16]
$Pd@Ir_{4L}/C$	0.1 M $HClO_4$	10.2	289	1.500	95%	[16]
$Li\text{-}IrO_x$	0.5 M H_2SO_4	50.0 (catalyst)	300	0.100 (@290mV)	_a	[17]
$IrNiO_x$	0.05 M H_2SO_4	10.2	N.A.	0.676	_a	[18]
$40\text{-}IrO_2/GCN$	0.5 M H_2SO_4	81.0 (IrO_2)	278	0.497 (@320mV)	95%	[19]
$Rh_{22}Ir_{78}/VXC$	0.5 M H_2SO_4	9.8	292	1.170	_a	[20]

IrW NDs	0.1 M $HClO_4$	10.2	300 (8.1 mA/cm^2)	0.794	95%	[21]
Au@Ir NRBs	0.5 M H_2SO_4	N.A.	296	0.694 (@320mV)	N.A.	[22]
Ru@IrO_x	0.05 M H_2SO_4	50.0 (catalyst)	282	0.650 (@320mV)	_a	[23]
$Pt_{62}Co_{23}/Ir_{15}$ FBNWs	0.1 M $HClO_4$	1.98	308	N.A.	N.A.	[24]
Ir_6Ag_9 NTs	0.5 M H_2SO_4	13.3	285	N.A.	95%	[25]
Ir_3Ag_9 NTs	0.5 M H_2SO_4	13.3	296	N.A.	95%	[25]
Au@$AuIr_2$	0.5 M H_2SO_4	20.0	261	1.44	90%	[26]
Au-Ir	0.1 M $HClO_4$	11.0	351	0.259	95%	[27]

[a] N.A. The related information was not given.

[b] _a. The *iR*-corrected was adopted for these references, but the detailed condition did not list in the literature.

Table S6 Detailed information of EIS fitting data.

The equivalent circuit used to fit the EIS data	Sample	R_s (Ω cm^2)	CPE-Yo (F/cm^2)	CPE-O (F/cm^2)	R_{ct} (Ω cm^2)
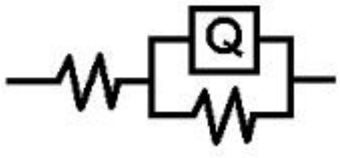	$Au@Au_{0.54}Ir_{0.46}$	4.79	7.34*10^{-3}	8.00*10^{-1}	21.27
	$Au@Au_{0.43}Ir_{0.57}$	4.69	4.46*10^{-3}	9.03*10^{-1}	24.26
	$Au@Au_{0.34}Ir_{0.66}$	4.73	2.56*10^{-3}	8.92*10^{-1}	31.03
	$Au@Au_{0.26}Ir_{0.74}$	4.32	2.66*10^{-3}	8.00*10^{-1}	36.48
	Au NPs	4.65	3.04*10^{-4}	8.74*10^{-1}	3187.00
	Ir/C	4.79	1.44*10^{-3}	8.90*10^{-1}	75.05

The ***ZSimpWin*** was used for the fitting of EIS data. The equivalent circuit used for the best fitting was shown in the table. The error of all fitting data is less than 3%.R_{ct} is the charge transfer resistance, R_s is internal resistance and CPE is double-layer capacitance.

Table S7 *d*-band center values of Au@Au_xIr_{1-x} and standard samples, obtained from valence band photoemission spectra.

Sample	*d*-band center position (eV)
Au@$Au_{0.54}Ir_{0.46}$	-4.43
Au@$Au_{0.43}Ir_{0.57}$	-4.44
Au@$Au_{0.34}Ir_{0.66}$	-4.37
Au@$Au_{0.26}Ir_{0.74}$	-4.27
Au NPs	-4.72
Ir powder	-3.99
IrO_2	-2.78

Table S8 *d*-band centers (ε_d) of Au_xIr_y studied by DFT calculations.

Sample	ε_d of Au_xIr_y (eV)	ε_d of Ir atom (eV)
Ir_9Au_{36}	-3.94	-2.40
$Ir_{18}Au_{27}$	-3.95	-3.27
$Ir_{27}Au_{18}$	-3.90	-3.46
$Ir_{36}Au_9$	-3.73	-3.57

Table S9 HER performances of $Au@Au_xIr_{1-x}$ compared with recently reported catalysts in acidic electrolytes.

Catalysts	Electrolyte	Mass loading ($\mu g/cm^2$)	η (mV) to 10 mA/cm^2	Tafel slope (mV/dec)	*iR*-corrected condition	Ref.
$Au@Au_{0.54}Ir_{0.46}$	0.5 M H_2SO_4	10.2 μg_{Ir}	27	14	90%	This work
$Au@Au_{0.43}Ir_{0.57}$	0.5 M H_2SO_4	10.2 μg_{Ir}	23	17	90%	This work
$Au@Au_{0.34}Ir_{0.66}$	0.5 M H_2SO_4	10.2 μg_{Ir}	31	17	90%	This work
$Au@Au_{0.26}Ir_{0.74}$	0.5 M H_2SO_4	10.2 μg_{Ir}	36	23	90%	This work
a-$RuTe_2$ PNRs	0.5 M H_2SO_4	200	33	35	95%	[28]
RuP_2@NPC	0.5 M H_2SO_4	1000	38	38	_a	[29]
Pt_1/OLC	0.5 M H_2SO_4	510	38	36	N.A.	[30]
Li-$IrSe_2$	0.5 M H_2SO_4	250	55	N.A.	100%	[31]
IrCoNi	0.1 M $HClO_4$	10.0 μg_{Ir}	33	32	95%	[32]
Ir/CC	0.5 M H_2SO_4	820	28	30	_a	[33]
Ru SAs@PN	0.5 M H_2SO_4	100 μg_{Ru}	24	38	_a	[34]
IrCo@NC-500	0.5 M H_2SO_4	285	24	23	N.A.	[35]
IrCo@NC-800	0.5 M H_2SO_4	285	52	74	N.A.	[35]
Ir_6Ag_9 NTs	0.5 M H_2SO_4	13.3	20	28	95%	[25]
Ir_3Ag_9 NTs	0.5 M H_2SO_4	13.3	34	33	95%	[25]

[a] N.A. The related information was not given.

[b] _a. The *iR*-corrected was adopted for these references, but the detailed condition did not list in the literature.

Table S10 Overall water splitting performances of Au@$Au_{0.43}Ir_{0.57}$ compared with recently reported Ir-based catalysts in acidic electrolytes.

Catalysts	Electrolyte	Mass loading (μg/cm^2)	Voltage (V) @10 mA/cm^2	*iR*-corrected condition	Ref.
Au@$Au_{0.43}Ir_{0.57}$	0.5 M H_2SO_4	20.0 μg_{Ir}	1.52	100%	This work
Ir/C‖Pt/C	0.5 M H_2SO_4	20.0 μg_{Ir}	1.63	100%	This work
Ir_6Ag_9 NTs/C	0.5 M H_2SO_4	13.3 μg_{Ir}	1.55	95%	[25]
Ir_3Ag_9 NTs/C	0.5 M H_2SO_4	13.3 μg_{Ir}	1.56	95%	[25]
IrCoNi	0.5 M H_2SO_4	N.A.	1.65	95%	[32]
Ir/GF	0.5 M H_2SO_4	820	1.55	_a	[33]
Pt/CC–Ir/CC	0.5 M H_2SO_4	820	1.65	_a	[33]
IrNi NCs	0.5 M H_2SO_4	N.A.	1.58	without	[36]

[a] N.A. The related information was not given.

[b] _a. The *iR*-corrected was adopted for these references, but the detailed condition did not list in the literature.

Table S11 Dissolved amount of metals from $Au@Au_{0.43}Ir_{0.57}$ and Ir/C catalysts in electrolyte after the durability test examined by ICP-OES.

Sample	Durability test	Dissolved Ir (μg)	Dissolved Au (μg)	Percentage of dissolved Ir from the initial mass	Percentage of dissolved Au from the initial mass
$Au@Au_{0.43}Ir_{0.57}$	10 mA/cm^2, 194 h	1.86	0	37.2 %	0
Ir/C	10 mA/cm^2, 1 h	1.26	_	25.2 %	_

Table S12 Overall water splitting durability of $Au@Au_{0.43}Ir_{0.57}$ compared with recently reported Ir-based catalysts in acidic electrolytes.

Catalysts	Electrolyte	Loading ($\mu g/cm^2$)	Current (mA/cm^2)/ Potential (V)	Time	Ref.
$Au@Au_{0.43}Ir_{0.57}$	0.5 M H_2SO_4	20.0 μg_{Ir}	10 mA/cm^2	194 h	This work
Ir/C || Pt/C	0.5 M H_2SO_4	20.0 μg_{Ir}	10 mA/cm^2	11 min	This work
CB[6]-Ir2	0.5 M H_2SO_4	20.0 μg_{Ir}	10 mA/cm^2	12 h	[37]
40-IG || Pt/C	0.5 M H_2SO_4	1000	1.6 V	24 h	[19]
IrW/C	0.5 M H_2SO_4	30.0 μg_{Ir}	10 mA/cm^2	8 h	[20]
IrNi NCs	0.5 M H_2SO_4	12.5 μg_{Ir}	1.6 V	10 h	[36]
Ir WNWs	0.1 M $HClO_4$	30.6 μg_{Ir}	10 mA/cm^2	11 h	[38]
Ir@N-G-750	0.5 M H_2SO_4	11.5 μg_{Ir}	20 mA/cm^2	40 h	[39]
RuIr-NC	0.05 M H_2SO_4	50.0	10 mA/cm^2	120 h	[40]
Ir-NSG	0.1 M $HClO_4$	73.3 μg_{Ir}	10 mA/cm^2	24 h	[41]
$Pt_{62}Co_{23}/Ir_{15}$ FBNWs	0.1 M $HClO_4$	40.0 μg_{Ir+Pt}	1.25 - 1.5 V ADT	10 k cycles	[11]
$Li\text{-}IrSe_2$	0.5 M H_2SO_4	3000	1.47 V	24 h	[31]

References

1. Yeh J, Lindau I. Atomic subshell photoionization cross sections and asymmetry parameters: $1 \leq Z \leq 103$. *Atomic Data and Nuclear Data Tables* 1985; **32**: 1.
2. Rudi S, Cui C, Gan L, et al. Comparative Study of the Electrocatalytically Active Surface Areas (ECSAs) of Pt Alloy Nanoparticles Evaluated by Hupd and CO-stripping voltammetry. *Electrocatalysis* 2014; **5**: 408-418.
3. Zhang G, Zhao D, Feng Y, et al. Catalytic Pt-on-Au nanostructures: why Pt becomes more active on smaller Au particles. *ACS nano* 2012; **6**: 2226-2236.
4. Kim D, Resasco J, Yu Y, et al. Synergistic geometric and electronic effects for electrochemical reduction of carbon dioxide using gold-copper bimetallic nanoparticles. *Nat. Commun* 2014; **5**: 4948.
5. Kresse G, Hafner J. ab-initio molecular-dynamics for open-shell transition-metals. *Phys. Rev. B* 1993; **48**: 13115-13118.
6. Kresse G, Furthmuller J. Efficient iterative schemes for ab initio total-energy calculations using a plane-wave basis set. *Phys. Rev. B* 1996; **54**: 11169-11186.
7. Kresse G, Furthmuller J. Efficiency of ab-initio total energy calculations for metals and semiconductors using a plane-wave basis set. *Comput. Mater. Sci.* 1996; **6**: 15-50.
8. Blochl P. Projector Augmented-wave Method. *Phys. Rev. B* 1994; **50**: 17953-17979.
9. Perdew J, Burke K, Ernzerhof M. Generalized gradient approximation made simple. *Phys. Rev. Lett* 1996; **77**: 3865-3868.
10. Vandersluis P, Spek A. BYPASS: an effective method for the refinement of crystal structures containing disordered solvent regions. *Acta Cryst* 1990; **46**: 194-201.
11. Hammer B, Nørskov J. Theoretical surface science and catalysis-Calculations and concepts. *Adv. Catal* 2000; **45**: 71-129.
12. Kim Y, Lim A, Kim J, et al. Highly efficient oxygen evolution reaction via facile bubble transport realized by three-dimensionally stack-printed catalysts. *Nat. Commun* 2020; **11**: 4921-4931.
13. Zhu J, Chen Z, Xie M, et al. Iridium-Based Cubic Nanocages with 1.1-nm-Thick Walls: A Highly Efficient and Durable Electrocatalyst for Water Oxidation in an Acidic Medium. *Angew. Chem. Int. Ed* 2019; **58**: 7244-7248.
14. Pi Y, Guo J, Shao Q, et al. Highly Efficient Acidic Oxygen Evolution Electrocatalysis Enabled by Porous Ir-Cu Nanocrystals with Three-Dimensional Electrocatalytic Surfaces. *Chem. Mater.* 2018; **30**: 8571-8578.

15. Seitz L, Dickens C, Nishio K, et al. A highly active and stable $IrO_x/SrIrO_3$ catalyst for the oxygen evolution reaction. *Science* 2016; **353**: 1011-1014.

16. Zhu J, Lyu Z, Chen Z, et al. Facile Synthesis and Characterization of $Pd@Ir_{nL}$ (n = 1-4) Core-Shell Nanocubes for Highly Efficient Oxygen Evolution in Acidic Media. *Chem. Mater* 2019; **31**: 5867-5875.

17. Gao J, Xu C, Hung S, et al. Breaking Long-Range Order in Iridium Oxide by Alkali Ion for Efficient Water Oxidation. *J. Am. Chem. Soc* 2019; **141**: 3014-3023.

18. Nong H, Reier T, Oh H, et al. A unique oxygen ligand environment facilitates water oxidation in hole-doped $IrNiO_x$ core-shell electrocatalysts. *Nat. Catal* 2018; **1**: 841-851.

19. Chen J, Cui P, Zhao G, et al. Low-Coordinate Iridium Oxide Confined on Graphitic Carbon Nitride for Highly Efficient Oxygen Evolution. *Angew. Chem. Int. Ed* 2019; **58**: 12540-12544.

20. Guo H, Fang Z, Li H, et al. Rational Design of Rhodium-Iridium Alloy Nanoparticles as Highly Active Catalysts for Acidic Oxygen Evolution. *ACS Nano* 2019; **13**: 13225-13234.

21. Lv F, Feng J, Wang K, et al. Iridium-Tungsten Alloy Nanodendrites as pH-Universal Water-Splitting Electrocatalysts. *ACS Cent. Sci.* 2018; **4**: 1244-1252.

22. Fan Z, Luo Z, Chen Y, et al. Synthesis of 4H/fcc-Au@M (M= Ir, Os, IrOs) Core-Shell Nanoribbons For Electrocatalytic Oxygen Evolution Reaction. *Small* 2016; **12**: 3908-3913.

23. Shan J, Guo C, Zhu Y, et al. Charge-Redistribution-Enhanced Nanocrystalline $Ru@IrO_x$ Electrocatalysts for Oxygen Evolution in Acidic Media. *Chem* 2019; **5**: 445-459.

24. Sun Y, Huang B, Li Y, et al. Trifunctional Fishbone-like PtCo/Ir Enables High-Performance Zinc–Air Batteries to Drive the Water-Splitting Catalysis. *Chem. Mater* 2019; **31**: 8136-8144.

25. Zhu M, Shao Q, Qian Y, et al. Superior overall water splitting electrocatalysis in acidic conditions enabled by bimetallic Ir-Ag nanotubes. *Nano Energy* 2019; **56**: 330-337.

26. Wang H, Chen Z, Wu D, et al. Significantly Enhanced Overall Water Splitting Performance by Partial Oxidation of Ir through Au Modification in Core-Shell Alloy Structure. *J. Am. Chem. Soc* 2021; **143**: 4639-4645.

27. Chen P, Li M, Jin S, et al. Heterostructured Au-Ir Catalysts for Enhanced Oxygen Evolution Reaction. *ACS Materials Lett* 2021; **3**: 1440-1447.

28. Wang J, Han L, Huang B, et al. Amorphization activated ruthenium-tellurium nanorods for efficient water splitting. *Nat. Commun* 2019; **10**: 5692.

29. Pu Z, Amiinu I, Kou Z, et al. RuP_2-Based Catalysts with Platinum-like Activity and Higher Durability for the

Hydrogen Evolution Reaction at All pH Values. *Angew. Chem. Int. Ed* 2017; **56**: 11559-11564.

30. Liu D, Li X, Chen S, et al. Atomically dispersed platinum supported on curved carbon supports for efficient electrocatalytic hydrogen evolution. *Nat. Energy* 2019; **4**: 512-518.
31. Zheng T, Shang C, He Z, et al. Intercalated Iridium Diselenide Electrocatalysts for Efficient pH-Universal Water Splitting. *Angew. Chem. Int. Ed* 2019; **131**: 14906-14911.
32. Feng J, Lv F, Zhang W, et al. Iridium-Based Multimetallic Porous Hollow Nanocrystals for Efficient Overall-Water-Splitting Catalysis. *Adv. Mater* 2017; **29**: 1703798.
33. Zhang J, Wang G, Liao Z, et al. Iridium nanoparticles anchored on 3D graphite foam as a bifunctional electrocatalyst for excellent overall water splitting in acidic solution. *Nano Energy* 2017; **40**: 27-33.
34. Yang J, Chen B, Liu X, et al. Efficient and robust hydrogen evolution: phosphorus nitride imide nanotubes as supports for anchoring single ruthenium sites. *Angew. Chem. Int. Ed* 2018; **130**: 9639-9644.
35. Jiang P, Chen J, Wang C, et al. Tuning the Activity of Carbon for Electrocatalytic Hydrogen Evolution via an Iridium-Cobalt Alloy Core Encapsulated in Nitrogen-Doped Carbon Cages. *Adv. Mater* 2018; **30**: 1705324.
36. Pi Y, Shao Q, Wang P, et al. General Formation of Monodisperse IrM (M = Ni, Co, Fe) Bimetallic Nanoclusters as Bifunctional Electrocatalysts for Acidic Overall Water Splitting. *Adv. Funct. Mater* 2017; **27**: 1700886.
37. You H, Wu D, Chen Z, et al. Highly active and stable water splitting in acidic media using a bifunctional iridium/cucurbit [6] uril catalyst. *ACS Energy Lett* 2019; **4**: 1301-1307.
38. Fu L, Yang F, Cheng G, et al. Ultrathin Ir nanowires as high-performance electrocatalysts for efficient water splitting in acidic media. *Nanoscale* 2018; **10**: 1892-1897.
39. Wu X, Feng B, Li W, et al. Metal-support interaction boosted electrocatalysis of ultrasmall iridium nanoparticles supported on nitrogen doped graphene for highly efficient water electrolysis in acidic and alkaline media. *Nano Energy* 2019; **62**: 117-126.
40. Wu D, Kusada K, Yoshioka S, et al. Efficient overall water splitting in acid with anisotropic metal nanosheets. *Nat. Commun* 2021; **12**: 1145.
41. Wang Q, Xu C, Liu W, et al. Coordination engineering of iridium nanocluster bifunctional electrocatalyst for highly efficient and pH-universal overall water splitting. *Nat. Commun* 2020; **11**: 4246.
